# An integrated edge AI prototype for smart agriculture: real-time pest detection, physical trapping, and multi-node deployment analysis under field uncertainty

**DOI:** 10.3389/fpls.2026.1853368

**Published:** 2026-06-23

**Authors:** Kunfu Wang, Shirong Guo, Xinyue Yang, Botao Lin, Weihua Cai, Rui Tang, Jianpu Lin

**Affiliations:** 1 School of Advanced Manufacturing, Fuzhou University, Quanzhou, China; 2 Faculty of Engineering, Monash University, Clayton, VIC, Australia; 3 The Hong Kong University of Science and Technology, Guangzhou, Guangdong, China

**Keywords:** chemical-free pest control, embedded ai, pest detection, pest monitoring and control, smart agriculture, YOLOv13

## Abstract

Timely pest management is crucial for minimizing crop losses and reducing chemical pesticide reliance in sustainable rice agriculture; however, continuous large-scale field monitoring remains labor-intensive and difficult to achieve. To advance integrated pest management, this study presents a solar-powered edge AI prototype for pest detection and trapping, evaluated through laboratory closed-loop testing and simulated multi-node deployment analysis, supplemented by preliminary field observation under outdoor conditions. A field-collected dataset comprising 1,500 images of 12 distinct rice pests from actual paddies in Fujian, China, was constructed for model training and performance assessment. In the perception module, a Mixed Aggregation backbone and a Ghost Convolution pre-head design are adopted to reduce computational cost while preserving accuracy. TensorRT-accelerated inference enables efficient on-device execution, and ESP32-based peripherals support real-time actuation and long-range communication in a closed-loop laboratory testbed under low power consumption. A cost-coverage deployment framework with a coverage-balanced greedy strategy is introduced for simulated placement analysis in fragmented paddy fields under varying resource budgets. Experimental results show that the proposed configuration achieves 96.1% mAP@0.5 and 59.4 FPS, corresponding to +0.7% accuracy and +19.6% speed gains over the YOLOv13 baseline. Under synthetic fog, rain, and low-light degradations, the selected model achieves the highest mAP in 6 of 9 scenarios, indicating favorable robustness trends under controlled perturbations. The embedded pipeline sustains 44.5 FPS on Jetson Xavier NX with 14.8 ms closed-loop control latency and 36.8 ms end-to-end response latency, and simulated multi-node deployment analysis indicates an average 1.6% coverage gain under identical device budgets while preserving full network connectivity. Preliminary outdoor trials only indicate that the system’s closed-loop workflow can operate under natural conditions and that trapping events were observed, serving as evidence of engineering feasibility; agronomic benefits still require further long-term quantitative evaluation.

## 1 Introduction

Agriculture is a fundamental pillar of national economic development, and its yield and quality are directly linked to food security and social stability (Fahad et al., 2021). Among the many factors affecting crop productivity, field pests represent a major threat, particularly in large-scale cultivation scenarios such as rice paddies. Pest outbreaks are often characterized by sudden onset and regional clustering, which makes timely and accurate monitoring essential (Fahad et al., 2021; Ma et al., 2025). Traditional pest monitoring methods typically rely on manual inspection or the post-identification of trapped insects using light traps (Kiritani, 1979). These methods are labor-intensive and difficult to sustain at scale. Consequently, the lack of real-time field data often forces farmers to rely on prophylactic or excessive chemical pesticide applications to prevent crop losses. This over-reliance disrupts the agroecological balance and severely contradicts the core principles of Integrated Pest Management (IPM). With the rapid advancement of smart agriculture, pest detection based on image recognition has emerged as a promising alternative to facilitate chemical-free IPM. However, the effectiveness of these approaches is often limited in complex outdoor environments due to varying lighting conditions, small and fast-moving targets, and cluttered backgrounds (Huihui et al., 2023). As a result, achieving a balance among detection accuracy, inference speed, and power efficiency to enable automated physical trapping remains a key challenge for practical deployment.

The process of pest identification and control in agricultural fields involves several interrelated stages, including detection, localization, trapping, and reporting (Kiritani, 1979; Qing et al., 2020, 2012). This integrated workflow poses three major technical challenges. First, pest detection is inherently a small-object recognition task. Pests typically occupy a very small number of pixels within natural background images and are frequently obscured by vegetation, soil texture, or lighting artifacts. Such conditions make it difficult for conventional detection algorithms to achieve both high precision and recall (Deng et al., 2022; Ebrahimi et al., 2017; Qiang et al., 2023; Rodríguez et al., 2021). Second, deployment environments impose strict resource constraints. Given the limited power supply, unstable connectivity, and large device density in agricultural settings, the deployed detection models must be highly lightweight and computationally efficient. These models should be capable of real-time inference on embedded platforms such as Jetson Xavier NX while maintaining low power consumption and operational reliability (Jia et al., 2023). Third, an integrated system that links detection with automated pest trapping remains under-discussed. Effective pest control systems must not only detect pests, but also coordinate actuator responses, such as spectral light control and fan triggering, while supporting remote communication and regional coordination. These requirements place high demands on system-level integration between software and hardware components, as well as on the design of responsive communication protocols (Qing et al., 2020, 2012).

Earlier approaches to pest detection primarily relied on traditional image processing techniques and handcrafted features, including color, texture, and edge descriptors, in combination with classical classifiers such as Support Vector Machines (SVM) (Ebrahimi et al., 2017; Rodríguez et al., 2021), K-Nearest Neighbors (KNN) (Kasinathan and Uyyala, 2021; Li and Ercisli, 2023), and Random Forests (Xu et al., 2021). For example, Rodríguez et al. used color thresholding and texture features to identify rice planthoppers and achieved an accuracy of 87.5% (Rodríguez et al., 2021). However, such methods are often highly sensitive to environmental variations and lack the generalization capability required for deployment in diverse field conditions. In recent years, deep learning-based object detection models have significantly improved the robustness and generalization performance of pest detection systems. Two-stage detectors such as Faster R-CNN (Deng et al., 2022) and SSD (Qiang et al., 2023) offer high detection accuracy, but their complex architectures and slow inference speed limit their usability in real-time field applications. One-stage detectors, particularly the YOLO (You Only Look Once) series from YOLOv3 to YOLOv13 (Jocher et al., 2020), have achieved a favorable balance between speed and accuracy and have been widely applied to crop pest detection tasks. For instance, Wang et al. proposed Insect-YOLO, which incorporates the CBAM attention mechanism (Woo et al., 2018) into the YOLOv8n backbone to improve detection performance on low-resolution field images, achieving an accuracy of 93.8% (Wang et al., 2025). Similarly, Huang et al. (2025) proposed YOLO-YSTs, an improved YOLOv10n detector that uses space-to-depth convolution and an inverted residual attention block to better detect small and overlapping pests on sticky-trap images while remaining light enough for edge inference. Nevertheless, most of these models rely on high-performance GPUs and are unsuitable for deployment on resource-constrained edge devices. To address the deployment bottleneck, researchers have explored lightweight neural networks such as MobileNet (Jia et al., 2023), ShuffleNet (Yang et al., 2021), and GhostNet (Peng et al., 2024). These architectures have been integrated with mainstream detection frameworks to develop compact models suitable for edge computing. While these efforts have yielded progress in balancing detection performance and computational efficiency, they often focus solely on algorithm-level optimization. Few studies have validated the complete detection pipeline under embedded conditions, which limits their direct applicability in real-world field scenarios.

Another emerging concern is the interpretability of deep learning models, which is particularly relevant in agricultural applications where model transparency is critical for reliability and system debugging. Various visualization techniques have been proposed to interpret model decisions. For example, Zhang et al. (2022) applied Gradient-weighted Class Activation Mapping (Grad-CAM) to visualize key features used by convolutional neural networks in the classification of fall armyworms. This method has since been widely adopted in pest detection studies to evaluate whether model attention aligns with insect body regions (Karim et al., 2024). However, Grad-CAM heavily depends on gradient backpropagation and is often rendered ineffective in lightweight networks due to inplace operations, pruning, or structural compression. Additionally, Grad-CAM is inherently class-specific and limited in visualizing multi-object attention or non-anchor-based detectors. These limitations reduce its reliability for visualizing model attention in practical embedded applications.

Simultaneously, the integration of embedded AI platforms with Internet of Things (IoT) technologies is becoming a key driver of intelligent agricultural systems (Kiobia et al., 2023). Devices such as Jetson Nano and Xavier NX offer low power consumption, high programmability, and on-board computing capabilities, making them suitable for on-field deployment of visual tasks (Doan and Phan, 2024; Zhang et al., 2023).

However, prior embedded efforts each address only one part of the problem. Doan and Phan (2024) built a Jetson-based pest recognition system but kept it at remote monitoring; Parameswari et al. (2021) focused on LoRa-based data transmission; and machine vision systems for rice light traps (Qing et al., 2020, 2012) automate the identification of trapped insects at the station side. None of these provides a single embedded system in which on-device detection, closed-loop physical trapping, and multi-node deployment operate together, which is the practical barrier to field use rather than detection accuracy alone.

In summary, although significant progress has been made in deep learning-based pest detection and agricultural IoT technologies (Kiobia et al., 2023; Wang et al., 2025; Zhang et al., 2022), several key challenges remain for real-world deployment. Most existing research emphasizes detection accuracy and lightweight model design but lacks a complete end-to-end workflow for embedded deployment, limiting practical applicability in complex field conditions. In addition, while some efforts have linked recognition with pest control devices, few have established a robust closed-loop system that supports real-time decision-making and dynamic regional coordination.

**Figure 1:**
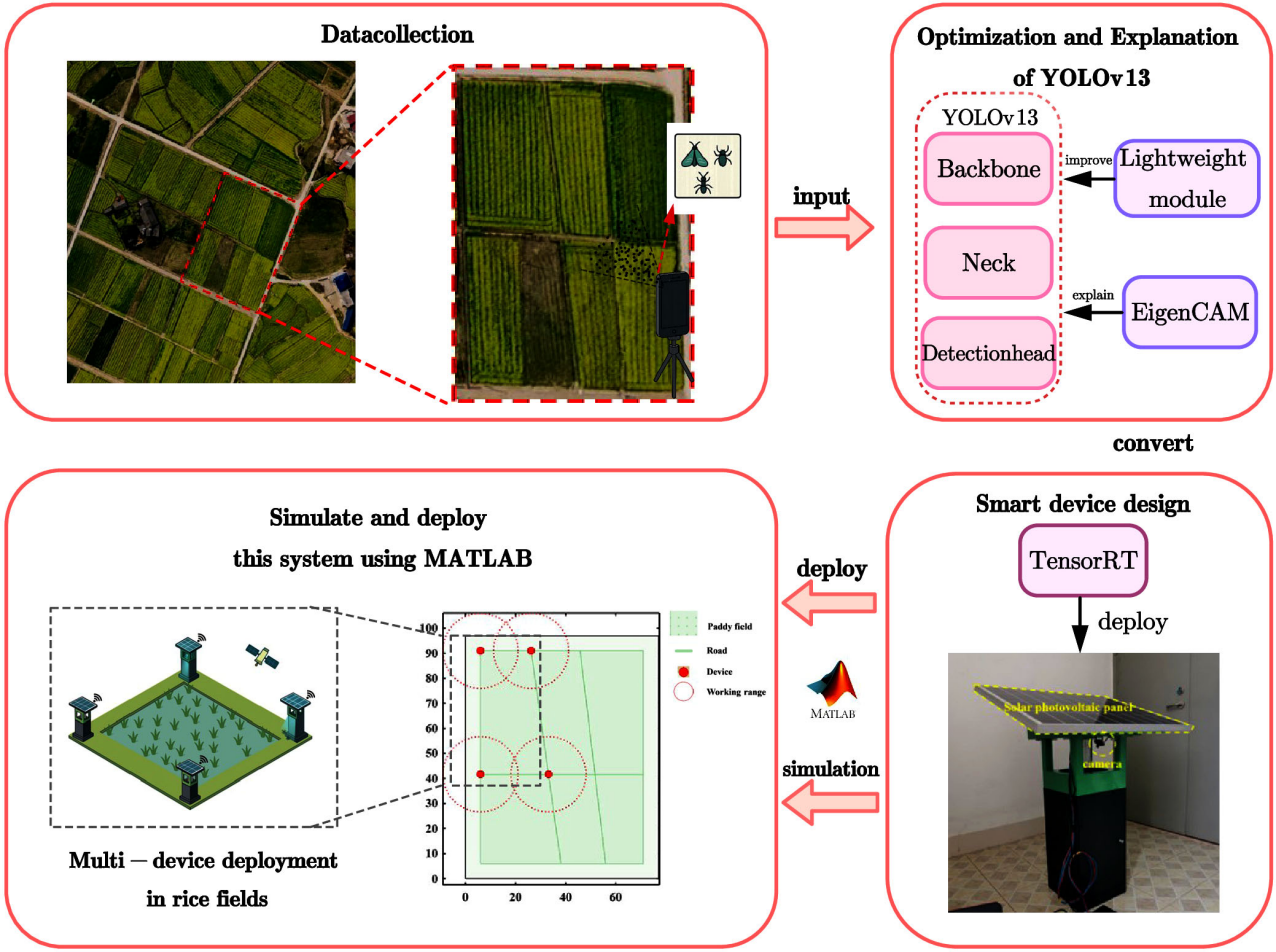
Framework of the proposed pest detection and trapping system.

To address these challenges, this study presents an integrated edge AI prototype that combines pest detection on the device, physical trapping in a closed loop, and deployment planning across multiple nodes into one working system, with the detector and the deployment strategy built to serve this system rather than offered as standalone advances. The proposed framework is built upon the YOLOv13 object detection architecture, incorporating two structural enhancements: a lightweight Mixed Aggregation Channel-split with 2-branch design (MAC2) module in the backbone, adapted from the mixed aggregation strategy in Mixed Aggregation Network (MANet), and Ghost Convolution (GhostConv) in the neck for improved efficiency in processing high-resolution features. In addition, EigenCAM is employed to visualize model attention, thereby enhancing interpretability and guiding architectural optimization. The trained model is integrated on a Jetson Xavier NX embedded testbed and connected to a custom hardware platform featuring pest-attracting light sources, fan-based physical trapping, Global Positioning System (GPS)-based localization, and LoRa-based remote communication. This hardware and software co-designed prototype is evaluated through laboratory closed-loop testing and simulated multi-node deployment analysis, using a cost and coverage planning formulation and a coverage balanced greedy strategy to guide placement, limit overlap, and preserve network connectivity. The complete workflow of data collection, model training, optimization, and feasibility evaluation is illustrated in **Figure 1**. The supporting contributions of this work are summarized as follows:

1. A field-collected pest image dataset covering 12 classes was constructed under complex rice-field conditions. All images were labeled and augmented, and additional synthetic degradations were generated for controlled robustness evaluation.
2. A YOLOv13-based detector was developed, integrating a lightweight MAC2 backbone module and GhostConv in the neck to reduce computational cost and raise throughput while preserving accuracy, with EigenCAM used to visualize activation maps and explain the design.
3. On-device detection, physical trapping, and LoRa reporting were integrated into a single TensorRT-accelerated embedded testbed, and end-to-end laboratory tests confirmed that this closed loop runs in real time and quantified its control and response latency.
4. A coverage-balanced greedy strategy was devised by incorporating spatial distribution and redundancy suppression into standard greedy selection. Conceptual benchmarking demonstrated its ability to reduce overlap, enhance coverage, and preserve full network connectivity while degrading gracefully under multi-node deployment uncertainty.

## 2 Materials and methods

The methodology of this study integrates object detection, hardware–software co-design, and field deployment modeling into a unified workflow. The complete process covers dataset construction, model development, system optimization, and feasibility verification. Each stage is closely related, forming a closed loop from data collection to laboratory and simulation evaluation.

### 2.1 System overview

**Figure 2:**
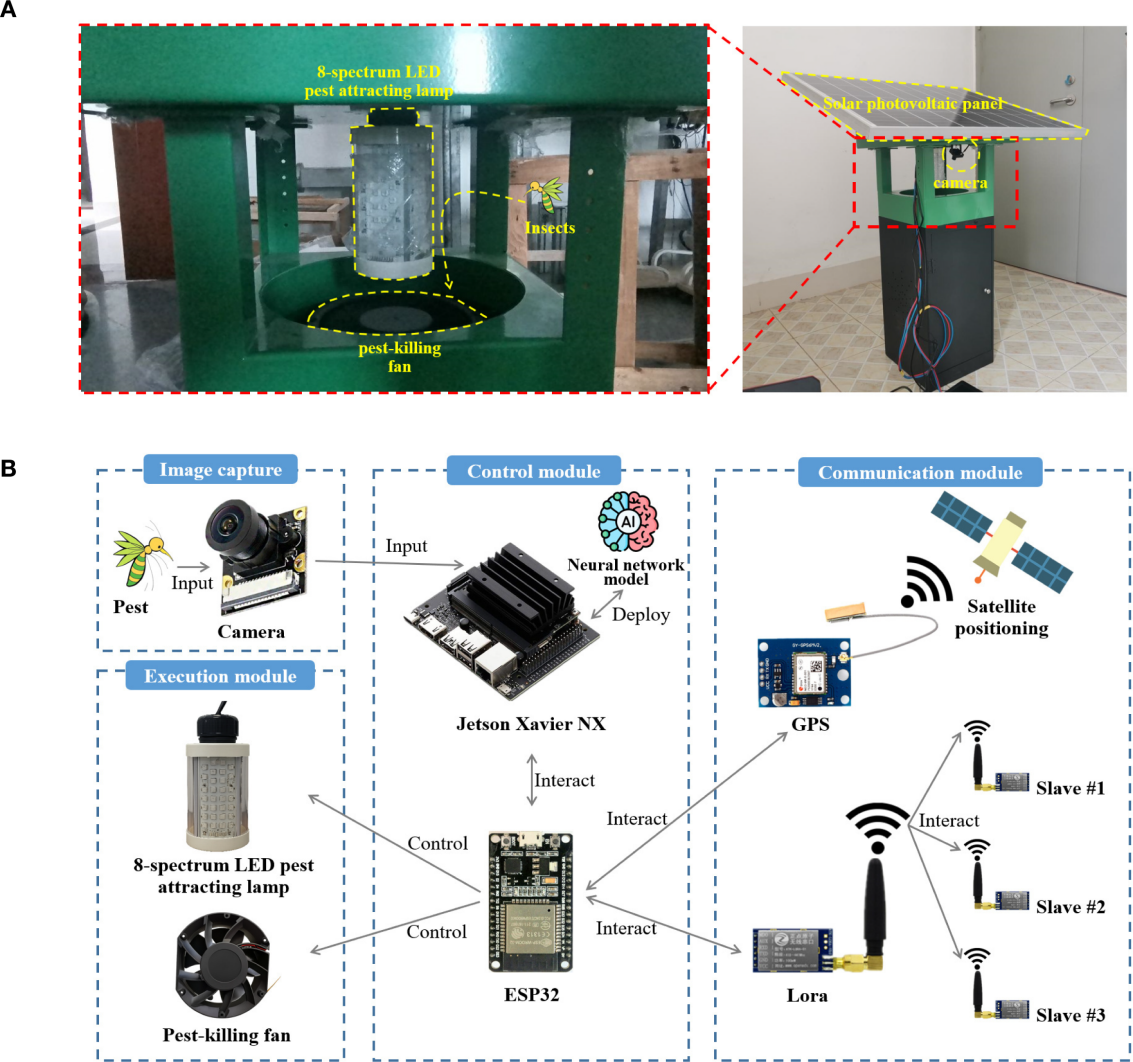
Smart pest-trapping device overview. **(A)** Device design. **(B)** Hardware structure.

Through the overall framework shown in **Figure 1**, the proposed system can be divided into two main components. The first component concerns the design and feasibility verification of a single autonomous prototype device, while the second focuses on the simulated optimization of multi-node cooperation in field deployment. The operational principle of a single device is illustrated in **Figure 2**. Each unit is equipped with a camera that continuously acquires pest images and transfers them to the embedded detection model. An eight-spectrum LED lamp generates light of specific wavelengths to attract insects, while a fan mechanism facilitates physical trapping once the insects approach the light source. The device is powered by a solar photovoltaic panel, ensuring autonomous and sustainable operation in outdoor environments without external energy supply (Cho et al., 2020). This configuration is intended to enable both pest recognition and physical suppression within a single platform.

**Figure 3:**
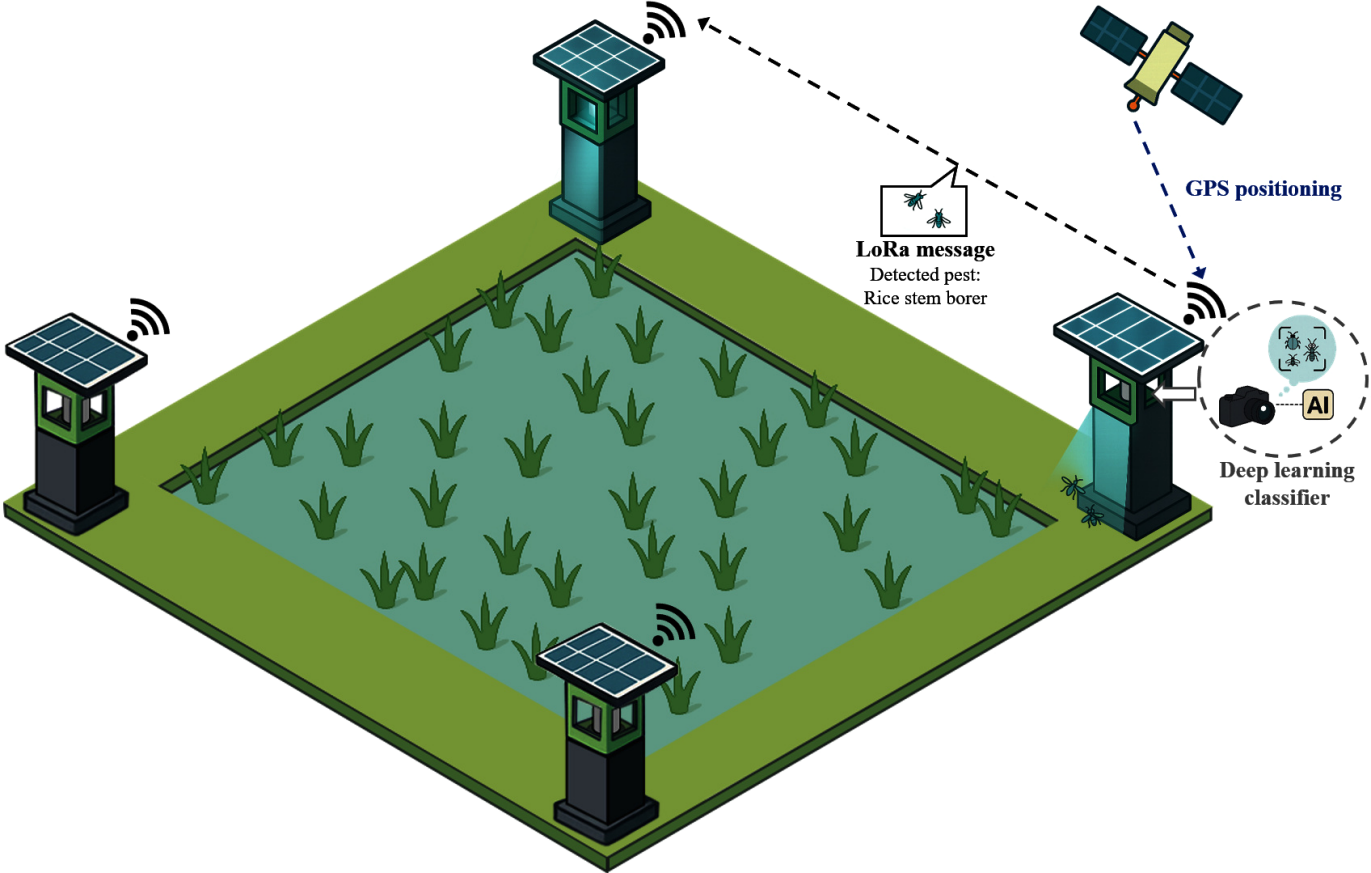
Field deployment of the smart devices.

The field deployment of multiple devices is demonstrated in **Figure 3**. Several units are distributed across the rice field and operate in a coordinated manner. Detected results are transmitted through LoRa communication to ensure low-power, long-range connectivity. GPS modules provide positional information, allowing detected pest events to be mapped to specific field locations. In addition to centralized monitoring, the transmitted results also enable device-to-device coordination. When one unit detects a dominant pest class, the information can be shared with neighboring devices, allowing them to pre-activate the corresponding light wavelength for targeted attraction. Through this collaborative mechanism, large-scale monitoring is complemented by adaptive responses, ensuring both detection coverage and efficient pest suppression in practical agricultural environments.

Based on the above overview, the following subsections provide the technical details of the system. Section 2.2 describes the design and deployment of the object detection model, including dataset preparation, architecture design, explainability analysis, and inference optimization. Section 2.3 then introduces the hardware prototype and software workflow used for feasibility testing, together with the simulated evaluation of multi-node cooperation in field deployment.

### 2.2 Method of pest detection

#### 2.2.1 Dataset construction and preprocessing

**Figure 4:**
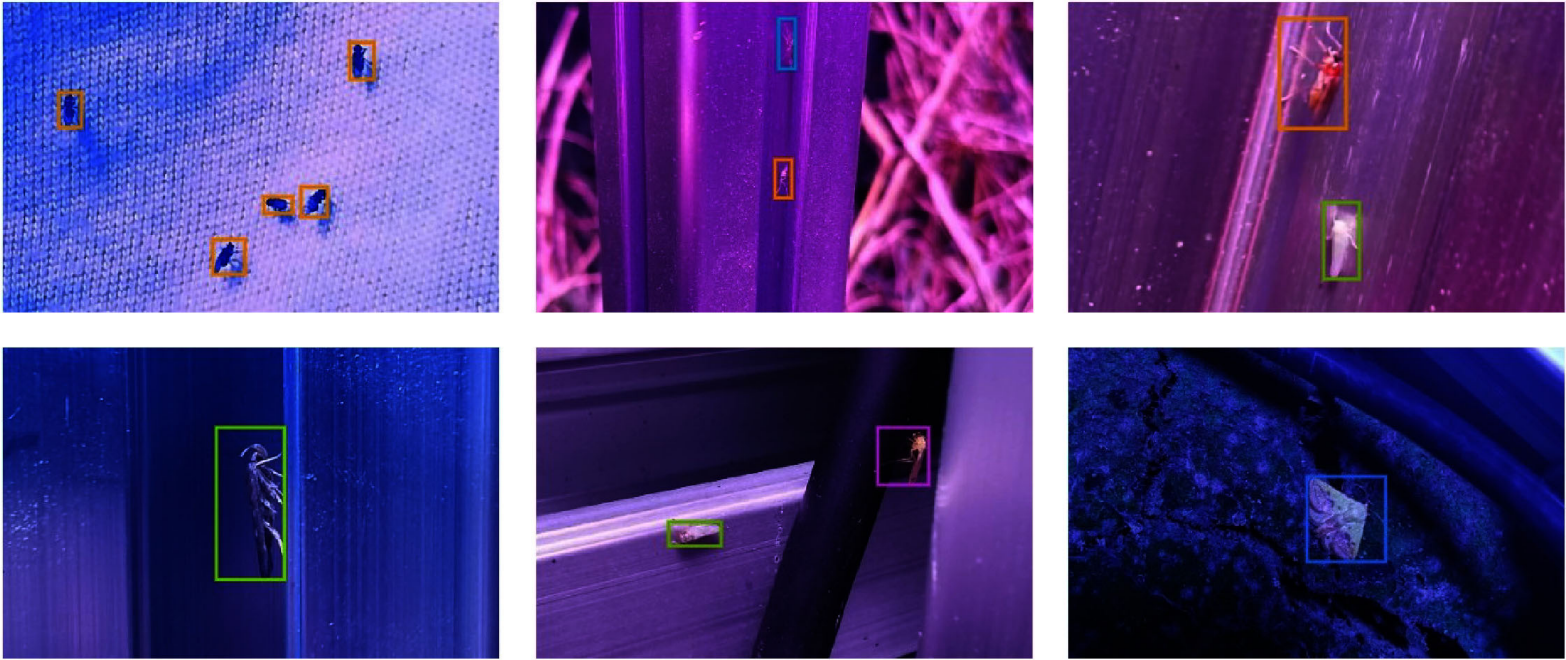
Overview of sample images in the pest detection dataset.

High-quality data acquisition and precise annotation are critical for pest detection in field environments, especially for small objects and visually similar insect species. Constructing a representative and well-annotated dataset is a prerequisite for training a high-performance detection model. Although annotated rice image datasets have been released for other tasks such as panicle analysis (Rashid et al., 2023), datasets dedicated to field pest detection remain limited. In this study, we collected 1500 original images during the summer season in real rice paddies at a farm in Jinjiang, Quanzhou, Fujian, China (118.67°E, 24.88°N). After quality and background screening, 1120 images were retained for annotation and partitioning. The dataset covers 12 common rice-field pest classes in southern China (Wenda-Piesik and Piesik, 2020): rice stem fly, rice gall midge, small brown planthopper, rice black bug, rice leaf bug, stem borer, leafhoppers (green and black-tailed types), rove beetle, longhorn beetle, rice thrips, brown planthopper, and leaf folder. As illustrated in **Figure 4**, all images were captured with a smartphone under natural field conditions with a uniform resolution of 1920 x 1200 pixels. These natural conditions span varied illumination, including dusk and low-light scenes, as well as partial occlusion by rice foliage. Real-world degraded samples are therefore present in both the training and evaluation subsets and contribute to the baseline numbers reported in Section 3.2.1.

All annotations were manually created using Roboflow. The 1120 retained images were partitioned at the original-image level into training, validation, and test subsets of 909, 152, and 59 images, respectively, so that no image or its augmented copies were shared across subsets; augmentation was applied only to the training set. Each original image was resized to a single 640×640 input. The complete dataset preprocessing workflow was conducted using Roboflow, which provided a streamlined process for image segmentation, annotation refinement, and format conversion. These preprocessing steps laid a reliable foundation for subsequent model training and evaluation, improving both model performance and generalization capability. The 909 training images were expanded threefold by zooming, rotation, and mosaic composition (Zhang et al., 2024), yielding 2,727 training images. The 152 validation and 59 test images were kept without augmentation. Consequently, the validation and test sets comprise 152 and 59 original images, respectively, each resized to a single 640×640 input. The 12 classes are heavily imbalanced and follow a long-tailed distribution: the most frequent class, Rice stem fly, alone accounts for 52.5% of all annotated instances, the two most frequent classes for 72.9%, and the four most frequent for 84.7%, whereas the six tail classes together contribute only about 7.5% (each about 2.5% or below). The per-class instance count ranges from 4757 down to 36 (a ratio of about 132:1), with a mean of 754 but a median of only 289 and a coefficient of variation of 1.72, indicating a strongly right-skewed distribution; the rarest class in this collection, Brown planthopper, contains only 36 instances. This imbalance reflects the field occurrence frequency observed during the present collection rather than a sampling or annotation artifact.

To further assess model behavior under controlled adverse visual perturbations, synthetic degradation was applied to the validation dataset using controlled image processing models. For fog simulation, we adopt a simplified atmospheric scattering model (He et al., 2010) defined as Equation 1:

(1) $$I\left(x\right)=J\left(x\right)\cdot t\left(x\right)+A\cdot \left(1-t\left(x\right)\right),t\left(x\right)=e^{-\beta d\left(x\right)}$$

Where *I*(*x*) is the observed foggy image, *J*(*x*) is the clean input image, *A* is the ambient light intensity (set as white), *β* controls the fog density, and *d*(*x*) is the normalized depth approximation simulated along the horizontal axis. The transmission map *t*(*x*) attenuates scene radiance and introduces an atmospheric veil.

Rain is synthesized by overlaying synthetic raindrop streaks using randomly sampled line segments with Gaussian blur smoothing. The rain-degraded image is expressed in Equation 2 (Zhang et al., 2019):

(2) I(x)=α·J(x)+(1−α)·R(x;ρ)

Where *R*(*x*) represents the synthetic rain layer and *α* is a blending coefficient controlling the transparency. The blending coefficient *α* is set to 0.3 so that streaks remained visible without saturating bright regions. In this model, level of rainfall intensity is simulated by adjusting the density parameter *ρ* of the streaks.

Low-light degradation is introduced by direct brightness suppression using a multiplicative attenuation model (Chen et al., 2018), as given in Equation 3:

(3) I(x)=γ·J(x),0<γ<1

Where *γ* is a brightness scaling factor corresponding to the severity level. All degraded images are generated offline and retain the same labels as the original dataset to ensure fair comparison across models.

This degradation framework provides a consistent and controllable testbed to benchmark the detection performance of lightweight models under selected adverse visual conditions. It partially approximates field imaging challenges, but does not replace validation in real environmental settings.

#### 2.2.2 Object detection model

**Figure 5:**
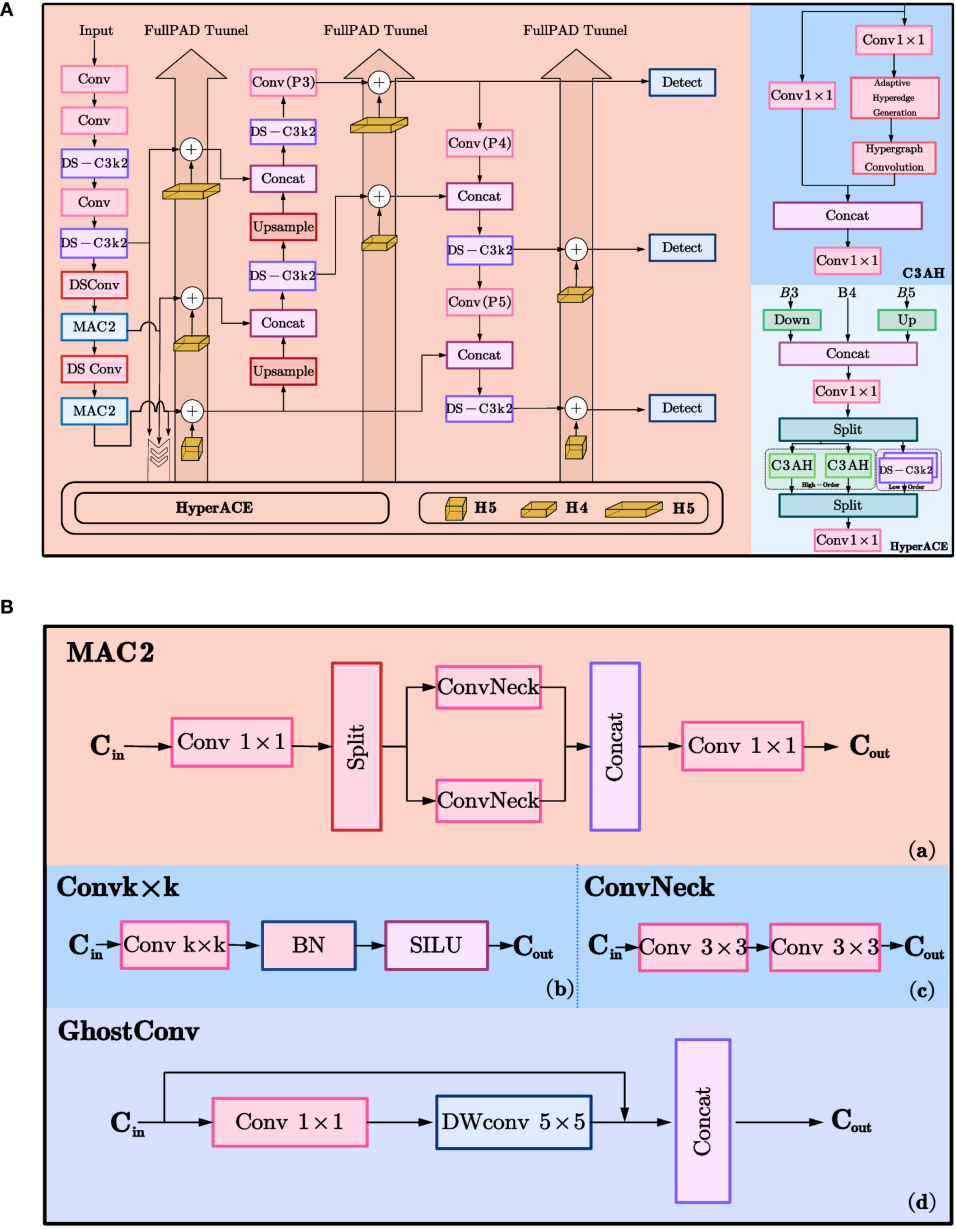
Improved YOLOv13 architecture and module details. **(a)** Overall architecture of the improved YOLOv13, including the MAC2 module for backbone and the pre-head Conv locations before the P3/P4/P5 detection heads where GhostConv is intended to replace the Conv. **(b)** Detailed illustration of MAC2 and GhostConv modules; internal labels (a–d) denote MAC2 dual-branch aggregation, the Conv block in one branch, ConvNeck in another branch, and GhostConv replacing the pre-head Conv before the P3/P4/P5 detection heads.

In real-world field pest detection tasks, the targets are typically small in size, irregular in shape, and often exhibit low inter-class variance (Ebrahimi et al., 2017). Moreover, the imaging conditions in agricultural environments are often degraded by fog, rain, or low illumination (Gao et al., 2020), which increases the difficulty of reliable feature extraction. To meet the practical requirements of accurate detection and real-time inference on resource-constrained embedded platforms, we redesign the baseline YOLOv13 architecture by incorporating two lightweight yet effective modules (as shown in **Figure 5**). Specifically, we adopt a Mixed Aggregation Channel-split backbone termed MAC2, adapted from the mixed-aggregation strategy in MANet, and apply the established GhostConv (Han et al., 2020) operator to the detection head. Both modules are existing techniques; the proposed design is therefore a task-specific integration tailored to the rice-pest detection setting rather than a fundamentally new detector building block. These improvements are aimed at achieving a better balance between detection accuracy, computational efficiency, and deployment feasibility on edge devices.

The MAC2 module, short for Mixed Aggregation Channel-split with 2-branch design, serves as a lightweight alternative to the A2C2f modules used in the original YOLOv13 architecture (Lei et al., 2025). While A2C2f provides rich multi-scale feature interaction, it is computationally expensive and unsuitable for edge deployment. Inspired by mixed aggregation strategies such as those in MANet (Feng et al., 2024), MAC2 adopts a dual-path residual structure that reduces computational complexity while retaining the ability to extract localized semantic features. As illustrated in **Figure 5b(a)**, given an input feature map **X** ∈ *R^C^*^×^*^H^*^×^*^W^*, the module first applies a 1x1 convolution to reduce the channel dimension to 2c: **X**_1_ = *Conv*_1×1_ (**X**). The resulting tensor **X**_1_ is evenly split along the channel axis into two parts: **F**_1_ and **F**_2_. The first part **F**_1_ is retained as-is to preserve original spatial features, while the second part **F**_2_ is processed through two independent transformation paths. The first path passes **F**_2_ through a bottleneck-like structure comprising a sequence of 1x1 depthwise (Chollet, 2017), and another 1x1 convolutional layer, as shown in **Figure 5b(b)**. The second path feeds **F**_2_ into a series of lightweight residual units referred to as ConvNeck modules (Feng et al., 2024), illustrated in **Figure 5b(c)**. Each ConvNeck module contains a pair of 3x3 convolutional layers with nonlinear activation in between, formally expressed in Equation 4:

(4) $$M_{k}\left(F_{2}\right)=\sigma \left(Conv_{3\times 3}^{\left(2\right)}\left(ReLU\left(Conv_{3\times 3}^{\left(1\right)}\left(F_{2}\right)\right)\right)\right)$$

Where *M_k_* denotes the *k*-th ConvNeck module, *σ* represents the final activation (e.g., ReLU (Daubechies et al., 2022)), and the superscripts (1) and (2) distinguish the two convolutional stages. The outputs of both transformation paths, along with the preserved **F**_1_, are concatenated along the channel dimension and fused by a final 1x1 convolution to form the output feature map in Equation 5:

(5) $$Y=Conv_{1\times 1}\left(Concat\left(F_{1},F_{2},M_{1}\left(F_{2}\right),\ldots ,M_{n}\left(F_{2}\right)\right)\right)$$

This structure not only maintains efficient spatial encoding and residual learning but also eliminates the need for attention mechanisms. The computational simplicity and representational diversity of MAC2 allow it to be effectively deployed on edge devices, while still capturing discriminative patterns essential for detecting small pests with weak texture contrast.

To improve real-time performance without altering the head itself, the pre-head convolution (the Conv immediately preceding the detection head) was modified. Specifically, on the P3 branch aimed at enhancing small-object detection, the conventional Conv before the head was replaced with a GhostConv module. For comparison, the same substitution was also applied to P4 and P5 in separate variants used for ablation and evaluation. As illustrated in **Figure 5b(d)**, GhostConv exploits redundancy in feature maps: a standard 3x3 convolution first generates a compact set of intrinsic features: $Y_{intr}=Conv_{3\times 3}\left(X\right)$. These are then passed through a depthwise convolution to produce additional ghost features: $Y_{ghost}=DWConv\left(Y_{intr}\right)$. The final output is obtained by concatenating both intrinsic and ghost components: $Y=Concat\left(Y_{intr},Y_{ghost}\right)$. This approach greatly reduces the number of parameters and floating-point operations, especially in the early detection stages where spatial dimensions are large and computational cost is high. We empirically observe that applying GhostConv exclusively to the P3 head yields the optimal trade-off between efficiency and detection performance. The deeper detection heads, P4 and P5, which operate on semantically abstract and lower-resolution feature maps, retain standard convolution layers to preserve their representational capacity.

In summary, the integration of MAC2 and GhostConv significantly enhances the lightweight design of the detection architecture, making it suitable for real-time pest detection tasks in constrained environments. These modules jointly improve both inference speed and detection precision, particularly for small and partially occluded pest targets. The proposed modifications lay the structural foundation for the subsequent system-level integration and performance evaluation.

#### 2.2.3 Model interpretability via visual attribution

To enhance the interpretability of the proposed detection model and verify the effectiveness of architectural modifications, we adopt EigenCAM (Muhammad and Yeasin, 2020), a gradient-free visual attribution method, to generate class activation maps. Unlike traditional gradient-based techniques such as Grad-CAM (Selvaraju et al., 2016), EigenCAM does not rely on backward propagation, making it highly suitable for modern architectures that involve extensive in-place operations or unstable gradients. This gradient independence ensures compatibility and stability during inference-time visualization, especially on embedded platforms where gradient computation may be impractical.

Given an input image *I*, let the output of a convolutional layer be a feature map *A* ∈ *R^C^*^×^*^H^*^×^*^W^*, where *C* is the number of channels, and *H* × *W* is the spatial dimension. EigenCAM first reshapes the feature map into a 2D matrix in Equation 6:

(6) $$A^{\prime }\in \;R^{C\times \left(H\cdot W\right)}$$

A covariance matrix is then computed from *A′*, and the first principal component **v**_1_ ∈ *R^C^* is extracted using principal component analysis (PCA). This component reflects the direction of maximum variance across channels. The class activation map *M*(*x,y*) is then obtained as a weighted sum of the original feature map channels in Equation 7:

(7) $$M\left(x,y\right)=\sum_{c=1}^{C}v_{1}^{\left(c\right)}\cdot A_{c}\left(x,y\right)$$

Where *A_c_*(*x,y*) is the activation at location (*x,y*) in channel *C*, and $v_{1}^{\left(c\right)}$ is the corresponding component in the first principal eigenvector. The resulting map *M* is normalized and resized to match the input resolution and can be overlaid on the original image for visualization. Unlike gradient-based methods like Grad-CAM, EigenCAM relies solely on forward features and does not require gradient computation with respect to class scores or loss functions. This makes it highly generalizable, stable, and particularly effective for inference-time visualizations in gradient-hostile networks.

#### 2.2.4 Module deployment and inference optimization

To enable practical deployment of the proposed detection model in agricultural field environments, the trained YOLOv13 model is first exported to the Open Neural Network Exchange (ONNX) (Jin et al., 2020) intermediate representation format. This standardization facilitates cross-platform inference and compatibility with hardware-accelerated runtimes. Subsequently, NVIDIA TensorRT (Jocher et al., 2022) is employed for model parsing, graph optimization, and inference acceleration, generating an efficient execution engine tailored for the Jetson Xavier NX platform.

**Figure 6:**
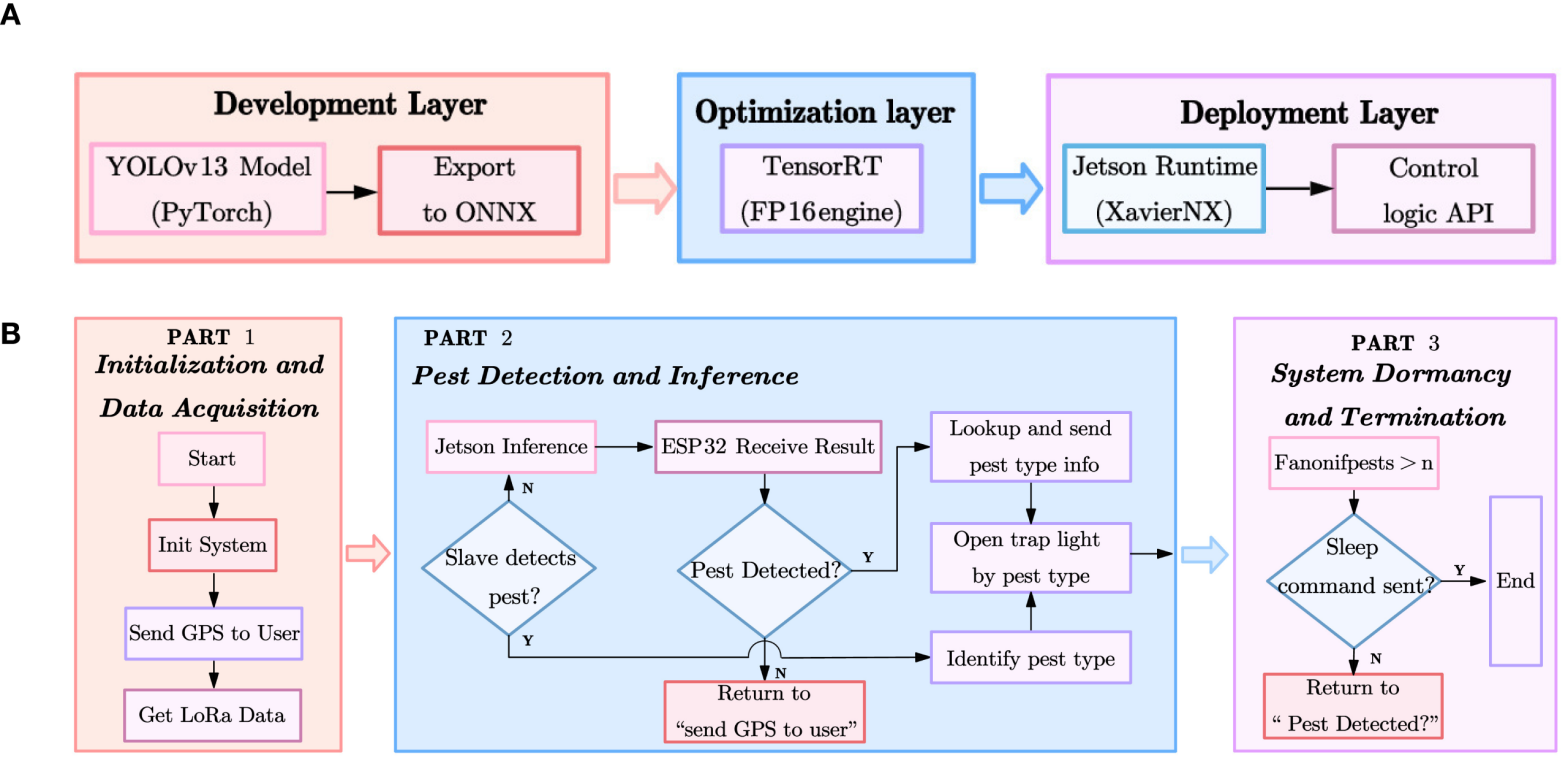
Deployment and runtime control workflow. **(a)** The trained YOLOv13 (PyTorch) model is exported to ONNX, built as an FP16 TensorRT engine, and executed on Jetson Xavier NX to produce detection outputs (class, bbox), which drive the control subsystem. **(b)** Monitoring and control logic from initialization and inference to actuation, communication, and low-power operation.

The deployment pipeline (**Figure 6a**) comprises three key stages. First, the PyTorch-trained model is exported to the ONNX format, preserving both the architectural structure and the learned weight parameters. Second, to reduce inference latency and computational load, FP16 (half-precision) quantization is applied (Jiang et al., 2018), maintaining acceptable accuracy while significantly improving runtime efficiency. Finally, the optimized TensorRT engine is integrated into the main control program through the TensorRT API, enabling real-time inference on video streams captured by onboard cameras. Once deployed, the model serves as the core perception module on the Jetson platform, continuously processing incoming image frames and outputting object categories and bounding box coordinates. These detection results are then transmitted to the control logic subsystem to trigger corresponding pest trapping responses. This deployment strategy ensures fast loading, low-latency inference, and stable long-term operation, providing a reliable, high-frequency perception backbone for intelligent pest control in embedded edge systems.

### 2.3 System design and deployment

To enable practical deployment of pest detection and trapping in field environments, a complete edge control system was developed based on the previously described lightweight detection model. The system is built upon the Jetson Xavier NX and ESP32 platforms and integrates LoRa communication, GPS positioning, and multispectral trapping modules to form a closed-loop workflow of detection, control, and communication with low power consumption. Furthermore, multi-node cooperative deployment in field scenarios is discussed, and a simulation model is designed to optimize the deployment strategy.

#### 2.3.1 Hardware design

To better understand the operational principle of the device, **Figure 2a** shows the assembled unit and **Figure 2b** illustrates the system architecture, which is divided into four functional submodules: image acquisition, control, execution, and communication. The image acquisition module continuously captures insect images through a camera, which are transmitted to the Jetson Xavier NX edge computing device for processing. A lightweight neural network deployed on the device performs real-time inference to identify pest species. The recognition results are delivered to the ESP32 microcontroller via a UART interface, enabling on-demand activation of the 8-channel spectral LED lamp and the pest-killing fan. This modular workflow integrates perception and actuation, forming a closed-loop detection-to-trapping mechanism that is well suited for embedded agricultural applications.

Therefore, the assembled device based on the proposed architecture is shown in **Figure 2a**. Inside the main housing, an 8-spectrum LED pest-attracting lamp is centrally positioned to emit light tuned to the visual sensitivity of multiple pest species (Shimoda and Honda, 2013). Below the lamp, a pest-killing fan is installed, activated upon detection events to physically remove insects. These components are controlled by an ESP32 microcontroller, which receives recognition results from the Jetson Xavier NX via a UART serial interface. The Jetson Xavier NX edge computing unit is housed in a sealed compartment at the lower section of the device. It is connected to a camera via a USB interface and communicates with the ESP32 through a dedicated serial port. The board runs the optimized object detection model and handles real-time inference. In terms of communication and positioning, a LoRa module is embedded within the ESP32 subsystem, supporting long-range star-topology networking with other terminal nodes. A GPS module is also connected via UART to provide real-time location data, which is periodically transmitted over the LoRa network to a central server. All internal components share a common power management system driven by the solar panel, regulated through a DC-DC converter and battery management circuit. This hardware configuration is intended to support outdoor deployment and to integrate recognition, actuation, and communication functionalities within a compact and modular enclosure.

#### 2.3.2 Software workflow for feasibility testing

The system software adopts a modular structure composed of a lightweight multithreaded architecture on the Jetson Xavier NX and a FreeRTOS-based task scheduling framework on the ESP32 (Guan et al., 2016), enabling efficient communication and real-time control in resource-constrained environments.

The detailed logic of the monitoring and control workflow is illustrated in **Figure 6b**. Upon power-up, the system performs hardware initialization and acquires GPS coordinates, which are sent to the central node for registration. Once initialized, the Jetson Xavier NX continuously performs pest detection based on incoming image streams. If no pest is detected, the system enters a low-power sleep state to conserve energy. When a pest is identified with sufficient confidence, its category is determined, and a structured recognition result is sent to the ESP32.

The ESP32 microcontroller parses the incoming data and performs a lookup to determine the corresponding LED spectral setting (Shimoda and Honda, 2013). The system then activates the appropriate light band to attract the pest, and if the detected pest count exceeds a predefined threshold, the fan is turned on to execute the trapping action. After control execution, the ESP32 constructs a JSON-formatted message that includes pest category, device status, and GPS coordinates, and transmits it via the LoRa network to the main node. This information is also used to update the regional control strategy. In the event of a low-activity state or command timeout, the system re-enters sleep mode to optimize power consumption. This logic ensures the entire system operates in a closed-loop manner: from real-time sensing and actuation to remote reporting and state management. Compared with traditional pest monitoring approaches that separate recognition, control, and communication into isolated subsystems, the proposed workflow achieves tight integration across modules. It enables faster response to pest events, real-time coordinated control across distributed nodes, and continuous spatial data acquisition for large-scale monitoring. Such an integrated design not only improves detection-to-response latency but also supports autonomous operation under low-power conditions, making the system highly suitable for long-term deployment in smart agriculture scenarios.

#### 2.3.3 Multi-node deployment in rice fields

**Figure 7:**
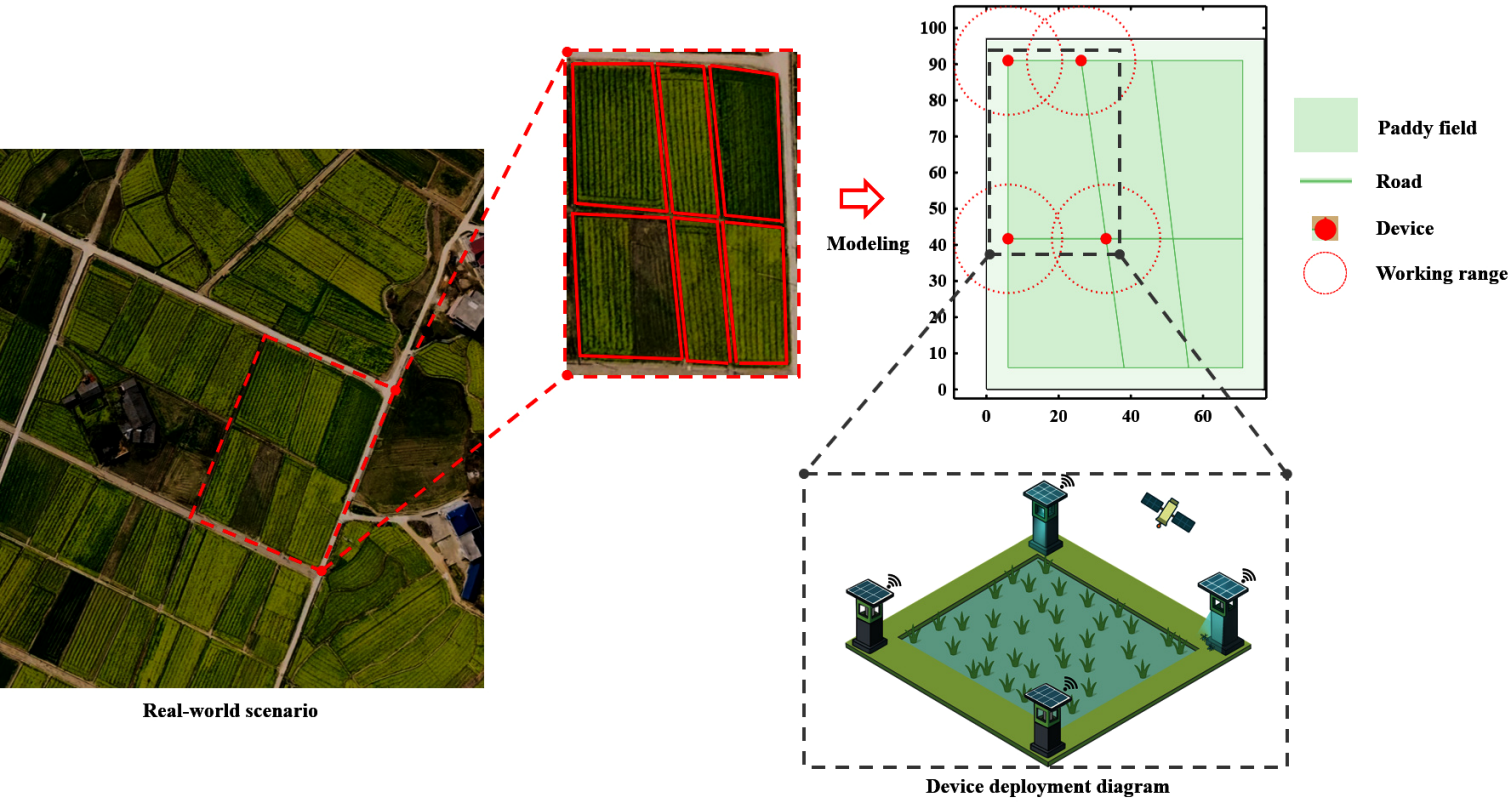
Schematic illustration of the paddy field scenario and device deployment.

To ensure that the proposed system can meet the requirements of large-scale rice field deployment, a field-level multi-node coordination simulation was conducted to explore optimal deployment strategies. Rice paddies in southern China are generally fragmented into small plots separated by ridges and narrow irrigation channels. Each plot was represented as a polygonal cell, and ridges were modeled as linear boundaries that constrain device placement. Candidate installation sites were generated along these ridges with fixed spacing, while the interior of each plot was discretized into a grid of sample points to evaluate coverage. The work range of the device was set to 15 m, corresponding to the maximum effective attraction range of the LED light source for pests (Prabaningrum and Moekasan, 2021). The communication radius was set to 200 m, reflecting the reliable transmission distance of LoRa modules under rural farmland conditions (Yim et al., 2018). To better approximate the characteristics of southern Chinese paddies, we modeled terraced paddies with widths of 10–20 m and lengths of 60–120 m (Hao et al., 2023). Reports from provincial and municipal agricultural surveys indicate that each household typically manages between 4.1 and 8.7 separate plots in this region, supporting the representativeness of this configuration (Ren et al., 2022). Road widths were set to 4 m based on national agricultural and land-use standards, which specify typical rural paths of 3–6 m (Hao et al., 2023). This abstraction allowed the deployment problem to be formulated as a coverage–connectivity optimization task under realistic field constraints. **Figure 7** shows the structural composition of the study area, including paddy fields (green), roads separating the plots (brown), and the outer boundary. Red dots indicate the deployed devices, each with a circular dashed line representing its effective working range.

The formulated problem can be categorized as a variant of the connected set cover problem, which is NP-hard and commonly addressed by heuristic or metaheuristic methods such as greedy algorithms, clustering-based strategies, or evolutionary search (Zangina et al., 2021). A greedy selection framework was therefore adopted in this study due to its computational efficiency and interpretability. In addition to the standard greedy (GR) selection, a cover-bridge greedy (CBG) strategy was introduced for this context.

Let *U* denote the set of grid points representing the interior of rice plots, and let *V* denote the candidate deployment sites along ridges. For each candidate site *v* ∈ *V*, its marginal coverage gain at iteration *t* is defined in Equation 8.

(8) $$g_{t}\left(v\right)=\left|\left\{u\in U\backslash C_{t-1}:d\left(u,v\right)\leq r_{w}\right\}\right|$$

where *C_t_*_−1_ is the set of already covered points after iteration *t*−1, and *r_w_* is the effective working radius of the LED attraction. In the standard greedy algorithm, the next site is selected according to Equation 9.

(9) $$v^{*}=\arg\max_{v\in V\backslash S_{t-1}}g_{t}\left(v\right)$$

where S*_t_*_−1_ is the set of already selected sites.

The proposed cover-bridge greedy strategy modifies this rule through a three-stage procedure. First, a seed set is constructed to increase coverage while avoiding redundant selections. At iteration *t*, a dispersion-aware score is defined in Equation 10.

(10) $$s_{t}\left(v\right)=g_{t}\left(v\right)+\lambda {\text{Δ}}_{t}\left(v\right)$$

Where $\Delta_{t}\left(v\right)=\min_{x\in S_{t-1}}d\left(v,x\right)$ with Δ*_t_*(*v*) = 0 if *S_t_*_−1_ = ∅, and *λ >* 0 is a weight factor. A fixed fraction of the budget is assigned to this seeding phase by repeatedly selecting according to Equation 11.

(11) $$v^{*}=\arg\max_{v\in V\backslash S_{t-1}}s_{t}\left(v\right)$$

Second, connectivity is enforced by bridging separate components formed by the seeded sites. Let *k_t_*_−1_ denote the set of connected components induced by *S_t_*_−1_ under the communication threshold *r_c_*. For each candidate *v*, let *b_t_*(*v*) be the number of distinct components in *k_t_*_−1_ that are adjacent to *v*. The bridging step selects according to Equation 12.

(12) $$v^{*}=\arg\max_{v\in V\backslash S_{t-1}}b_{t}\left(v\right)\cdot s_{t}\left(v\right)$$

with lexicographic preference given to a larger *b_t_*(*v*) and *s_t_*(*v*) used as a tiebreaker. This step is repeated until a single connected component is obtained or the budget is exhausted.

Third, the remaining budget is used to improve coverage while preserving connectivity. Candidates adjacent to the current connected set are ranked by *b_t_*(*v*), and the top-scoring site is added at each iteration. If no adjacent candidate exists, the nearest site to the current set is chosen to restore adjacency and ranking by *s_t_*(*v*) then continues.

By prioritizing dispersed high-gain seeds, the initialization phase reduces coverage redundancy. By selecting bridge sites that touch multiple components, connectivity is achieved with few additional devices. By filling only with adjacent candidates ranked by *s_t_*(*v*), connectivity is preserved while coverage increases. Simulation experiments were conducted by varying the number of devices to generate cost–coverage curves under equal spacing, random connected placement, standard greedy, and cover-bridge greedy. Each trial produced coverage ratios and connectivity measures averaged across repeated runs for the stochastic strategy. To avoid an overidealized assessment, the deployment model was additionally evaluated under injected operational uncertainty whose parameters were taken directly from the hardware measurements in Section 3.3: device localization error was modeled as a zero-mean Gaussian offset with *σ* = 5 m, each communication link was made available only with probability 1 − *p* with *p* = 0.012, and each device was independently rendered inoperative with probability 0.053. For every device budget, 300 Monte Carlo trials were run, and coverage and connectivity were reported as mean ± standard deviation in Section 3.4. The chosen parameters, such as field size, ridge width, device spacing, and communication thresholds, followed reported agronomic data and field deployment guidelines (Prabaningrum and Moekasan, 2021; Ren et al., 2022; Yim et al., 2018). This ensured that the modeling results provided a practical basis for evaluating deployment efficiency. The outcomes of these simulations are analyzed in Section 3.4.

## 3 Results

To evaluate the technical performance of the proposed integrated pest detection and trapping system under controlled experimental settings, experiments were conducted from three perspectives: object detection accuracy and inference speed, embedded system responsiveness, and the overall linkage effectiveness of pest trapping. This section presents experimental design, baseline comparisons, and evaluation metrics, followed by a detailed analysis of key performance outcomes.

### 3.1 Experimental settings and evaluation metrics

All experiments in this study were conducted using a custom pest image dataset collected and annotated from rice fields at a farm in Jinjiang, Quanzhou, Fujian Province. The dataset was partitioned at the original-image level into 909 training, 152 validation, and 59 test images before any resizing or augmentation, so that no image or its augmented copies appeared in different subsets. After resizing each image to 640×640 and applying threefold augmentation to the training set only, training used 2727 images (the 909 training images expanded threefold), with 152 and 59 unaugmented images for validation and test. The models were trained for up to 300 epochs with a batch size of 16, using the SGD optimizer with default settings (Lei et al., 2025). Model development and testing were implemented in Python based on the PyTorch 1.11.0 framework and executed on an NVIDIA Tesla T4 GPU with 16 GB of memory. Final deployment and inference evaluations were performed on an NVIDIA Jetson Xavier NX edge device. Unless otherwise stated, all reported detection metrics in this work are obtained from a single training run with a fixed random seed; statistical reporting across repeated runs was not performed. For the cross-architecture comparison in Section 3.2.1, the baseline detectors YOLOv5n, YOLOv8n, YOLOv10n, YOLOv12n, YOLOv13n, PicoDet-m, and Insect-YOLO were each instantiated from their published default configurations and trained under the same protocol described above: the same dataset partition, the same SGD optimizer with default settings, 300 epochs, batch size 16, and a fixed random seed. No per-model hyperparameter search was performed, so the reported numbers reflect each architecture’s out-of-the-box behaviour on this dataset rather than per-model optimisation effort.

In this study, five evaluation metrics were employed to comprehensively assess the performance of the pest detection module: Precision, Recall, mean Average Precision (mAP), inference time, and model size. The first three metrics are standard in the object detection domain and are defined as follows: Equations 13–15 define the three metrics used in this study:

(13) $$P\left(k\right)=\;\frac{TP}{TP+FP}\times 100\%$$

(14) $$R\left(k\right)=\;\frac{TP}{TP+FN}\times 100\%$$

(15) $$mAP=\;\frac{1}{C_{s}}\sum_{M=i}^{N}P\left(k\right)\text{Δ}R\left(k\right)$$

where *TP* is the number of true positives, *FP* is the number of false positives, and *FN* is the number of false negatives. *C_s_* denotes the number of object categories, and *N* is the number of interpolated recall points used for *mAP* calculation. *P*(*k*) and Δ*R*(*k*) represent the precision and the increment in recall at the *k*-th threshold, respectively. Precision quantifies the accuracy of positive predictions, while Recall evaluates the model’s ability to detect all relevant instances. The *mAP* aggregates performance across all categories and IoU thresholds, offering a single, comprehensive metric for object detection accuracy. In addition to detection accuracy, inference time (ms) and model size (MB) were used to evaluate deployment efficiency on edge devices. Together, these metrics provide a balanced assessment of both detection performance and practical use for real-time pest monitoring tasks in the field.

From an agronomic decision perspective, these metrics are not only algorithmic indicators. False alarms can increase unnecessary interventions (extra trap activation, additional field checks, and avoidable energy use), while missed detections can delay response and increase outbreak risk. Therefore, model operating points should be selected to balance intervention cost and prevention benefit in practical crop protection workflows.

### 3.2 Comparative evaluation of pest detection methods

This section evaluates the proposed detection framework through four aspects. Section 3.2.1 compares representative lightweight architectures to establish a performance baseline. Section 3.2.2 analyzes the impact of introducing the MAC2 backbone and GhostConv modules. Section 3.2.3 examines model robustness under synthetic visual degradations. Section 3.2.4 investigates model interpretability using CAM-based visualization. These experiments comprehensively assess detection accuracy, comparative stress-test behavior, and explainability.

#### 3.2.1 Comparison of detection architectures

**Table 1** summarizes the quantitative performance of six detection models evaluated on the same pest dataset using the PyTorch framework. The metrics include mean average precision at IoU 0.5 (mAP@0.5), precision, recall, average inference time per image, and model storage size. All models were tested using consistent input resolution and inference batch size to ensure comparability. The selected models include YOLOv5n, YOLOv8n, YOLOv10n, YOLOv12n, YOLOv13n (baseline) (Lei et al., 2025), and PicoDet-m (Yu et al., 2021), a high-efficiency architecture optimized for edge deployment. In addition, Insect-YOLO, a pest-specific detection algorithm, is also included for comparison (Wang et al., 2025). YOLOv13n incorporates structural improvements over previous iterations, while maintaining lightweight characteristics compatible with embedded systems.

**Table 1:** Performance comparison of object detection models under the PyTorch framework.

Model	mAP@0.5	Precision	Recall	Inference (ms)	Size (MB)
YOLOv13n	95.4	92.3	84.5	10.7	5.2
YOLOv12n	85.8	88.4	77.0	9.6	5.5
YOLOv10n	89.4	86.5	77.7	4.8	5.8
YOLOv8n	93.3	88.5	86.7	5.2	6.0
YOLOv5n	88.9	80.5	80.6	6.5	5.3
PicoDet-m	90.5	91.3	76.5	7.2	4.9
Insect-YOLO	91.2	84.3	82.4	5.5	6.1

From the results in **Table 1**, YOLOv13n achieves the best overall detection performance, with a mAP@0.5 of 95.4%, precision of 92.3%, and recall of 84.5%. This represents notable improvements over previous YOLO versions. Compared to YOLOv12n and YOLOv10n, YOLOv13n improves mAP by 9.6 and 6.0 percentage points, respectively. In terms of recall, YOLOv13n outperforms YOLOv5n and YOLOv10n by approximately 4.0% and 6.8%, indicating better detection coverage of target instances. Although YOLOv8n achieves slightly higher recall (86.7%), its mAP (93.3%) and precision (88.5%) are still lower than YOLOv13n, while also having a larger model size of 6.0 MB. In terms of inference latency, YOLOv13n requires 10.7 ms per frame, which is slightly higher than earlier YOLO versions (YOLOv10n: 4.8 ms, YOLOv8n: 5.2 ms) and PicoDet-m (7.2 ms). This is the expected trade-off for achieving higher accuracy.

Notably, despite its competitive precision (91.3%), PicoDet-m suffers from low recall (76.5%), indicating it may miss a large number of pest targets in complex environments. In addition, Insect-YOLO does not show clear advantages on this dataset and even underperforms YOLOv8n in overall detection accuracy. Overall, YOLOv13n achieves the best balance between detection accuracy and efficiency, offering a strong baseline for further architectural enhancements in subsequent experiments.

#### 3.2.2 Ablation study on module improvements

To evaluate the effectiveness of the proposed architectural modules in our detection framework, we conducted ablation experiments focusing on two key enhancements: how the placement of GhostConv influences the model performance when used in conjunction with MAC2. The results are provided in **Table 2**, where five models are compared: the baseline YOLOv13 without any enhancements, YOLOv13-M with only MAC2, and three variants of MAC2-enhanced models with GhostConv inserted at different feature pyramid levels, specifically at P3 only, at both P3 and P4, and at P3, P4, and P5 simultaneously. This design allows us to isolate and analyze the individual and combined effects of the MAC2 and GhostConv modules, as well as their sensitivity to the depth of the feature pyramid hierarchy.

**Table 2:** Ablation study of MAC2 and GhostConv enhancements in the YOLOv13 detection framework.

Model	MAC2	GhostConv	mAP@0.5	Precision	Recall	Inference (ms)	Size (MB)	Cos-sim	HF-ratio
YOLOv13	x	x	95.4	92.3	84.5	10.71	5.19	0.217	0.185
YOLOv13-M	✓	x	95.8	91.5	85.2	8.30	5.99	0.239	0.162
YOLOv13-M-G-P3	✓	P3	96.1	92.7	88.8	6.95	5.98	0.304	0.145
YOLOv13-M-G-P34	✓	P3 + P4	96.2	88.7	86.1	8.40	5.96	0.290	0.136
YOLOv13-M-G-P345	✓	P3 + P4 + P5	92.9	87.5	87.3	9.34	5.82	0.311	0.135

From **Table 2**, several key insights emerge. Firstly, integrating MAC2 alone (YOLOv13-M) leads to noticeable performance improvements compared to the baseline YOLOv13, raising the mAP@0.5 from 95.4% to 95.8%, and recall from 84.5% to 85.2%, with only a modest increase in model size (from 5.19 MB to 5.99 MB). This confirms the effectiveness of MAC2 in enhancing feature representation. Secondly, inserting GhostConv (Han et al., 2020) at the P3 level on top of MAC2 (YOLOv13-M-G-P3) brings further improvements across all key metrics: mAP@0.5 reaches 96.1%, precision rises to 92.7%, and recall increases to 88.8%. These gains are likely due to the edge-preserving and information-condensing properties of GhostConv, which are particularly beneficial at low-level feature maps like P3, where fine spatial detail is crucial. However, further expansion of GhostConv to deeper levels results in diminishing or even negative returns. The YOLOv13-M-G-P34 model (GhostConv at P3 + P4) achieves a slightly higher mAP (96.2%) but at the cost of reduced precision (88.7%) and recall (86.1%). The YOLOv13-M-G-P345 model, with GhostConv applied at all three levels, suffers a clear drop in mAP (92.9%) and precision (87.5%), although recall remains moderately high (87.3%). To directly examine the mechanism underlying this degradation, we probed the feature map fed into the shallow detection head (P3) for each of the five trained variants on the full 152-image validation set, and report two statistics in **Table 2**: (i) the mean absolute pairwise channel cosine similarity (Cos-sim), which increases with channel redundancy, and (ii) the high-frequency 2-D FFT energy ratio (HF-ratio), which decreases under over-smoothing. The channel redundancy at P3 rises from 0.217 (baseline YOLOv13) to 0.311 (YOLOv13-M-G-P345), an overall +43% increase that is largely monotonic in the depth of GhostConv placement, while the high-frequency energy decreases strictly monotonically from 0.185 to 0.135 (−27%). Taken together, these statistics indicate that GhostConv insertion progressively accumulates channel redundancy while reducing high-frequency content across the feature pyramid, with the signal concentrated at the shallow P3 head that matches the small-pest regime dominating this dataset. Detection accuracy absorbs this degradation through two-level (P3+P4) replacement but drops sharply at the three-level configuration (mAP@0.5 from 96.2% to 92.9%), consistent with the cumulative loss of representational diversity at the shallow head exceeding what the detection head can compensate.

Together, the ablation experiments clearly demonstrate the utility of both proposed modules, with nuanced observations. The MAC2 backbone proves to be an effective lightweight feature extractor that enhances both accuracy and efficiency, outperforming the deeper MAC2f variant. The GhostConv module is most beneficial when applied selectively, with its greatest impact observed at the P3 level, where spatial detail is critical. Overapplication of GhostConv to multiple pyramid levels is associated with increased channel redundancy and reduced high-frequency content at the shallow P3 head (**Table 2**), consistent with the feature over-smoothing and interference mechanisms reported in prior work (Lu et al., 2022). Therefore, the best-performing configuration in terms of accuracy, model size, and inference speed is the YOLOv13-M-G-P3 model, which is adopted as the final version for deployment in subsequent experiments.

#### 3.2.3 Robustness under synthetic visual degradations

**Figure 8:**
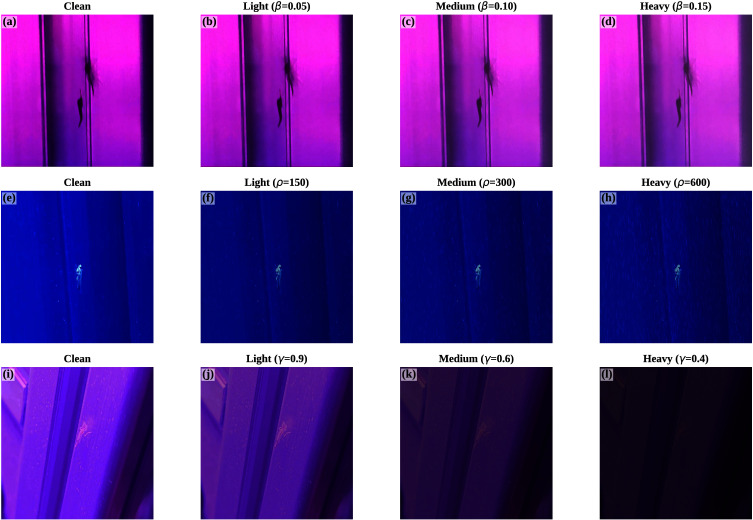
Examples of synthetically generated image degradation under three conditions: **(a–d)** fog with scattering coefficient *β* = 0.05/0.10/0.15; **(e–h)** rain with droplet density *ρ* = 150/300/600; **(i–l)** dark environments with *γ* = 0.9/0.6/0.4. The “Clean” column shows the original image without degradation.

Because real-world degradations vary continuously and uncontrollably across illumination, weather, and occlusion conditions, synthetic perturbations are used in this subsection to enable a controlled, parameterized stress test in which all model variants are compared on identical noise scenarios. Performance on naturally degraded samples already contributes to the baseline mAP reported in Section 3.2.1; the present subsection isolates the per-perturbation sensitivity that controlled synthesis makes measurable. To evaluate model robustness under controlled synthetic degradations, we conducted experiments simulating three representative types of visual degradation, namely fog, rain, and low-light environments (Gao et al., 2020). As shown in **Figure 8**, each type of degradation includes three severity levels: light, medium, and heavy. For fog, the scattering coefficient *β* in Equation 1 is set to 0.05, 0.10, and 0.15 (He et al., 2010). For rain, the droplet density *ρ* in Equation 2 is set to 150, 300, and 600 (Zhang et al., 2019). For dark environments, the brightness scaling factor *γ* in Equation 3 is adjusted to 0.9, 0.6, and 0.4 (Chen et al., 2018). These ranges follow established practice in synthetic-degradation benchmarks, where multiple severity levels characterise model behaviour under controlled corruptions (Hendrycks and Dietterich, 2019), and they bracket the operating envelope of rice-paddy imaging. For fog, *β* from 0.05 to 0.15 maps, via the Koschmieder relation *V* ≈ 3.912*/β*, to atmospheric visibilities of about 78 m down to 26 m, spanning light morning mist to heavy paddy fog. For rain, *ρ* from 150 to 600 spans the low-to-high streak densities of the synthetic rain pipeline in (Zhang et al., 2019). For low light, *γ* from 0.9 to 0.4 corresponds, under the multiplicative attenuation model above, to retained brightness of 90% down to 40%, spanning dusk to deep low-light. The same per-severity parameter set was applied uniformly to every validation image, with no per-image randomisation or sampling. The leftmost column in each row displays the original clean image without any degradation and serves as the visual reference. These degraded versions are then used to examine comparative performance trends under varying perturbation types and severities.

**Figure 9:**
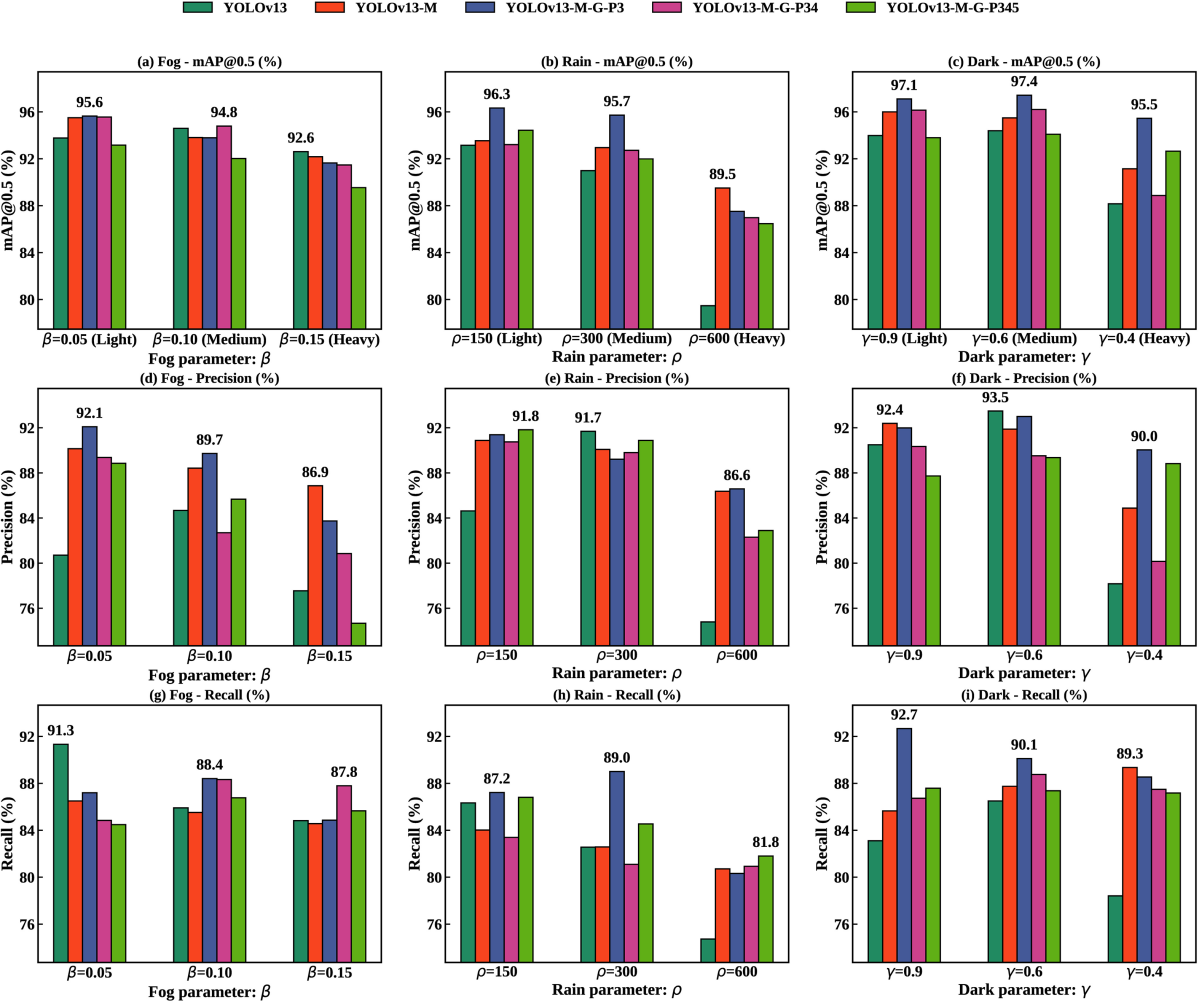
mAP@0.5 (%), precision (%), and recall (%) of five models (YOLOv13 and its variants) under three synthetic degradation scenarios: **(a–c)** mAP@0.5 under Fog, Rain, and Dark conditions; **(d–f)** Precision under Fog, Rain, and Dark conditions; and **(g–i)** Recall under Fog, Rain, and Dark conditions. Each scenario includes light, medium, and heavy degradation levels.

**Figure 9** illustrates variations in mAP@0.5, precision, and recall for all models under three visual degradation types and three severity levels. The subplots are arranged such that the first column corresponds to fog, the second column to rain, and the third column to dark environments. The top row (a–c) presents mAP@0.5, the middle row (d–f) shows precision, and the bottom row (g–i) shows recall, each under light, medium, and heavy degradation. Precision indicates the ability to avoid false detections, which is important for reducing over-spraying and false alarms. mAP@0.5 reflects the combined quality of localization and classification. Recall measures the ability to detect all targets, which is critical because missed pests can trigger uncontrolled outbreaks. Taken together, the three metrics describe relative detection reliability under synthetic visual degradation settings.

For mAP@0.5 (**Figures 9a–c**), a consistent comparative performance trend is observed across degradation types. Under fog, YOLOv13-M-G-P3 performs best at the light level (95.6%), while YOLOv13-M-G-P34 leads at the medium level (94.8%). Under heavy fog, the baseline YOLOv13 reaches 92.6%, which is 2.0% higher than YOLOv13-M-G-P3 (91.6%), indicating that simpler architectures can better preserve coarse localization when high-frequency details are suppressed. In rain, YOLOv13-M-G-P3 ranks first at both light and medium severities (96.3% and 95.7%), outperforming the baseline by 6–8%. However, at heavy severity, YOLOv13-M slightly exceeds YOLOv13-M-G-P3 (89.5% vs 87.5%), likely because the MAC2 backbone balances feature resolution and semantic aggregation and better mitigates occlusion noise. In dark environments, YOLOv13-M-G-P3 maintains the top score across all severities (97.1%, 97.4%, and 95.5%), clearly outperforming YOLOv13 (93.9%, 94.4%, and 88.2%) and suggesting that early GhostConv at P3 improves boundary recovery under low contrast in this controlled test setting.

For precision (**Figures 9d–f**), precision decreases as severity increases, although the drop varies by model. In fog, YOLOv13-M-G-P3 achieves the highest precision at light (92.1%) and medium (89.7%) levels, corresponding to relative improvements of +11.4% and +5.0% over YOLOv13 (80.7%, 84.7%). Under heavy fog, YOLOv13-M slightly exceeds YOLOv13-M-G-P3 (86.9% vs 86.4%), which implies that GhostConv at P3 benefits moderate blur, whereas the simpler MAC2 backbone generalizes better under extreme scattering. In rain, YOLOv13-M-G-P345 is best at light severity (91.8%) and is marginally higher than YOLOv13-M-G-P3 (91.4%); at medium severity, YOLOv13-M-G-P3 reaches 89.2%; under heavy rain, it is similar to YOLOv13-M (86.4%). The baseline falls to 74.8% at heavy rain, lagging the top variants by 12–14 points. In dark conditions, YOLOv13-M performs best at light severity (92.4%), whereas YOLOv13-M-G-P3 is best at medium and heavy severities (93.0% and 90.0%). These results indicate that GhostConv at P3 improves resilience to contrast loss in synthetic tests, while deeper insertions at P34 or P345 provide limited benefit and are accompanied by increased channel redundancy and reduced high-frequency content at P3 (**Table 2**), consistent with the over-smoothing reported in prior work (Li et al., 2023).

For recall (**Figures 9g–i**), recall also decreases with severity, and the identity of the best model varies more than for precision or mAP. In fog, YOLOv13-M-G-P3 is best at medium (88.4%), whereas YOLOv13-M-G-P34 is best at heavy (87.8%). Under light fog, the baseline YOLOv13 reaches 91.3%, which suggests that excessive architectural changes can reduce sensitivity in mildly noisy backgrounds (Dodge and Karam, 2017). In rain, YOLOv13-M-G-P3 is best at light and medium severities (87.2% and 89.0%), whereas YOLOv13-M-G-P345 is best at heavy severity (86.8%); multilevel GhostConv likely compensates for partial occlusions but can increase false positives. In dark scenes, YOLOv13-M-G-P3 maintains high recall at light and medium severities (92.7% and 90.1%), while YOLOv13-M is slightly higher at heavy severity (89.4%). Proper placement of GhostConv therefore helps preserve recall under low contrast in synthetic degradations (Li et al., 2023), whereas simpler structures can avoid overfitting to local background noise.

Overall, YOLOv13-M-G-P3 shows strong relative robustness under synthetic degradations, achieving top-1 performance in 6 out of 9 mAP conditions, 5 out of 9 precision cases, and 5 out of 9 recall cases (**Table 3**). However, it is not always the best. Models like YOLOv13-M-G-P34 and YOLOv13-M-G-P345 demonstrate advantages under severe fog or rain, while YOLOv13-M occasionally outperforms in dark or heavily occluded environments. The baseline model even surpasses modified variants under light degradation in some recall cases. These observations indicate that GhostConv at P3 improves early feature discrimination in controlled perturbation tests, but excessive modification may reduce generalizability. Because these experiments are based on offline synthetic perturbations applied to clean images, they should be interpreted as comparative stress tests rather than direct evidence of robustness in real paddy environments involving combined weather effects, motion blur, backlighting, occlusion, or lens contamination. For field deployment, model selection should therefore balance synthetic stress-test performance with simplicity, and further validation on independently collected real-environment datasets remains necessary.

**Table 3:** Number of top-1 performance rankings across all degradation conditions.

Model variant	mAP@0.5 (top-1)	Precision (top-1)	Recall (top-1)	Total
YOLOv13-M-G-P3	6	5	5	16
YOLOv13-M	1	3	1	5
YOLOv13	1	0	1	2
YOLOv13-M-G-P34	1	0	1	2
YOLOv13-M-G-P345	0	1	1	2

In practical farm protection, precision-oriented settings reduce unnecessary interventions, while recall-oriented settings reduce missed early infestation signals. This enables managers to choose conservative operating points during high-risk outbreak windows and cost-aware operating points during stable periods.

#### 3.2.4 CAM-based interpretability analysis

**Figure 10:**
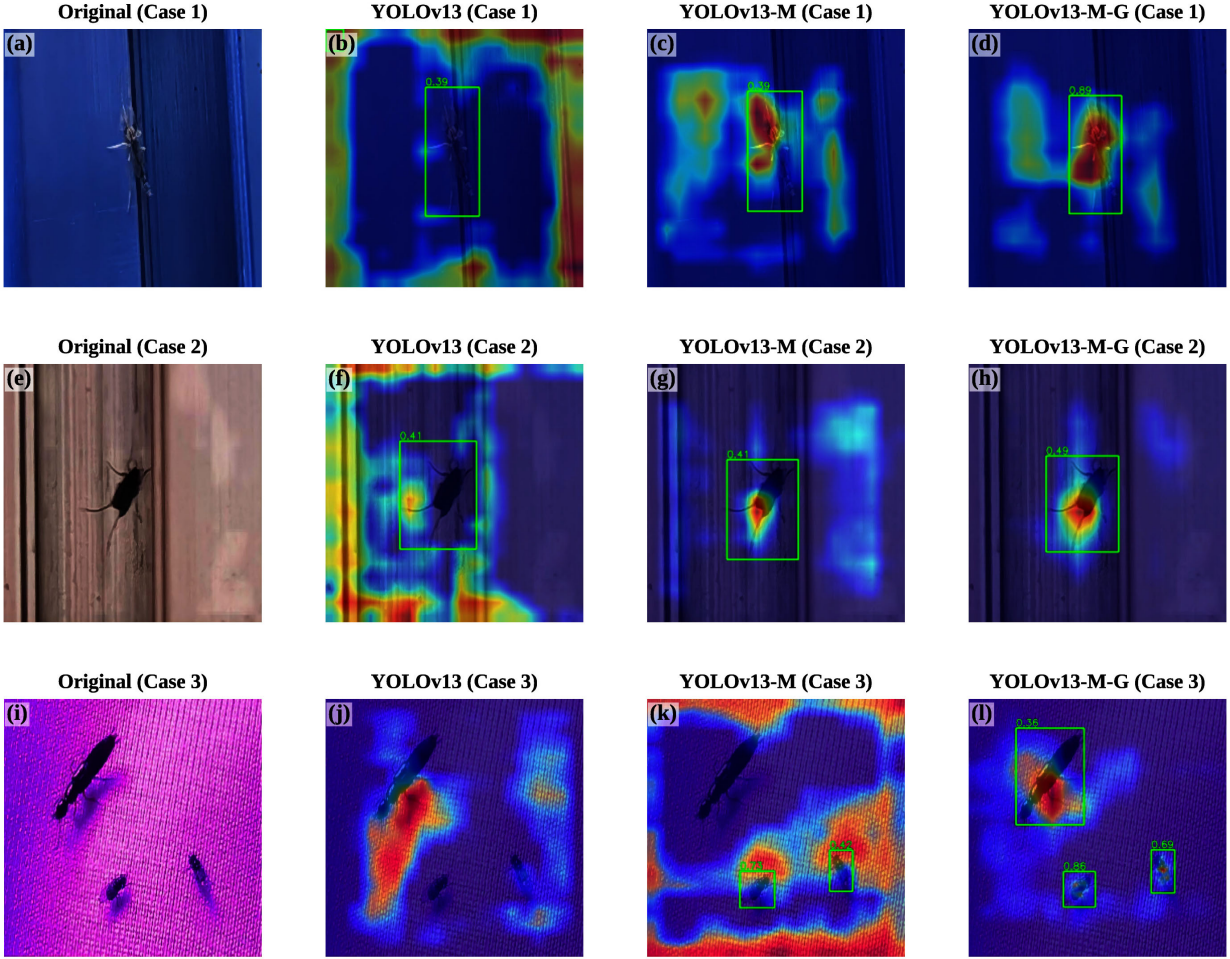
EigenCAM visualizations of three representative insect detection cases. Columns correspond to the original images **(a, e, i)**, YOLOv13 **(b, f, j)**, YOLOv13-M **(c, g, k)**, and YOLOv13-M-G **(d, h, l)**. Green bounding boxes indicate detection results.

To further investigate the internal mechanisms underlying the performance differences among the proposed models, we conducted an interpretability analysis based on class activation mapping. Specifically, we utilized EigenCAM (Muhammad and Yeasin, 2020) to visualize the spatial attention responses of three representative models: YOLOv13, YOLOv13-M, and YOLOv13-M-G (equivalent to YOLOv13-M-G-P3; abbreviated as YOLOv13-M-G hereafter). These models were evaluated on three typical insect detection cases that involve visual challenges such as low contrast, partial occlusion, and multiple object interference. As shown in **Figures 10a–l**, the green bounding boxes indicate the predicted detection regions, while the overlaid color heatmaps represent the model’s internal attention distribution with respect to spatial importance. These visualizations provide intuitive insights into how each model perceives insect features and how the proposed architectural components influence focus accuracy and interpretability.

From the visualization results in **Figures 10b, f, j**, the baseline YOLOv13 demonstrates relatively dispersed and unfocused attention patterns. In Case 1, although the primary insect target is roughly covered, the attention region also spreads significantly to background textures, which may introduce false activations under real-world clutter. In Case 2, the model highlights broad areas surrounding the insect but fails to concentrate on the critical thorax or wings, which are often key anatomical indicators for pest identification. In Case 3, YOLOv13 fails to detect any insect instances, indicating its limited capability in handling visually noisy backgrounds and multiple small objects simultaneously.

As seen in **Figures 10c, g, k**, the YOLOv13-M model demonstrates a clearer and more condensed focus on the insect targets. With the incorporation of the MAC2 module, the model forms more coherent attention maps (Feng et al., 2024). In Case 1, the activation begins to concentrate more around the pest body, though it still includes background interference. In Case 2, the model highlights the central thorax area more distinctly, showing improvement in semantic localization. In Case 3, YOLOv13-M enhances attention toward small insect targets; however, its focus on the larger pest is insufficient, leading to missed detection. These observations indicate that MAC2 contributes to improving the semantic aggregation capability of the network but still lacks robustness in balancing attention across objects of different scales.

The YOLOv13-M-G model, shown in **Figures 10d, h, l**, presents the most refined attention maps. In Case 1, the attention is sharply confined to the pest’s contour, with minimal distraction from surrounding textures. In Case 2, the model focuses almost exclusively on the insect’s head and thorax, enhancing interpretability by emphasizing regions with high recognition value. In Case 3, YOLOv13-M-G successfully identifies all insect instances, including both the large pest and smaller occluded ones, with localized and independent activation zones. This result suggests that the addition of GhostConv at the P3 feature level strengthens the model’s sensitivity to fine structural details, enabling more precise localization in multi-object scenarios.

Overall, the interpretability analysis using EigenCAM confirms that the proposed architectural enhancements improve not only quantitative performance but also the model’s ability to produce human-understandable decisions. The YOLOv13-M-G model demonstrates clearer attention behavior and more reliable focus areas across all three cases, validating its effectiveness in complex pest detection tasks. These visualizations should be read as qualitative attribution evidence for the plausibility of the model’s attention, not as rigorous proof of biological correctness.

### 3.3 System-level testing

To verify the real-time capability and system integration performance of the proposed pest detection and trapping framework, we conducted a series of hardware-in-the-loop tests under laboratory conditions using two working nodes. These tests aimed to evaluate the inference efficiency of the detection models, the responsiveness of the closed-loop control chain, and the communication stability between subsystems.

First, inference speed was benchmarked for five detection models using two execution frameworks: PyTorch (FP32) and TensorRT (FP16) (Jocher et al., 2022). As shown in **Table 4**, in the PyTorch environment (FP32), the baseline YOLOv13 model achieved 23.87 frames per second (FPS), while YOLOv13-M and YOLOv13-M-G-P3 reached 31.71 and 31.72 FPS respectively, demonstrating the benefit of the lightweight MAC2 backbone. In the TensorRT-accelerated deployment (FP16), the FPS significantly improved across all models. Notably, YOLOv13-M-G-P34 achieved the highest inference speed of 60.49 FPS, followed by YOLOv13-M-G-P3 at 59.40 FPS. These runtime differences arise from (i) the reduced convolutional load of the MAC2 backbone, (ii) the placement of GhostConv, which yields greater acceleration at P3 under FP32 due to larger feature maps, and (iii) TensorRT’s FP16 kernel fusion and Tensor Core scheduling, which better exploit the layer configuration of the P34 variant. These results support the feasibility of the proposed architectural enhancements for edge deployment on embedded platforms such as the Jetson series.

**Table 4:** FPS of object detection models under PyTorch (FP32) and TensorRT (FP16)^1^.

Metric	YOLOv13	YOLOv13-M	YOLOv13-M-G-P3	YOLOv13-M-G-P34	YOLOv13-M-G-P345
FP32 FPS	23.87	31.71	31.72	29.98	31.48
FP16 FPS	49.68	58.30	59.40	60.49	59.15

^1^Tested on NVIDIA Tesla T4 GPU.

In the system-level testing, a laboratory closed-loop experiment was conducted to evaluate real-time performance and coordination. Static pest images were presented to the camera, and detection results were transmitted via UART to an ESP32 microcontroller, which interpreted the class output and triggered the corresponding actuator (multispectral LED or fan). The average communication delay from Jetson to ESP32 was 4.3 ms, and the ESP32 control logic plus relay activation added 7.6 ms. Together with other system overheads, this yielded a 14.8 ms closed-loop latency. Including inference, the end-to-end latency from image acquisition to actuator response averaged 36.8 ms; under this pipeline, the detector sustained 44.5 FPS on Jetson Xavier NX. To further assess stability, a 60-minute endurance run was performed with two independent nodes and approximately 150 image presentations. The system produced 142 successful responses, corresponding to a 94.7% laboratory closed-loop response success rate. The remaining cases were primarily associated with borderline-confidence predictions, suboptimal illumination, or occasional control delays exceeding tolerance thresholds. In the laboratory experiment, a response was scored as successful only when the actuator triggered matched the predicted class, so classification errors within the 12 pest classes contribute to this residual 5.3% failure rate alongside the detection-side issues noted above. In actual field operation this metric is conservative: because the fan provides a class-agnostic physical trapping mechanism, opening it whenever any pest is detected would still trap insects in the cases where only the species prediction was wrong. The fan therefore acts as an engineering redundancy that absorbs classification-level errors at the actuation stage, and the practical trapping success rate is expected to exceed the strict 94.7% reported here. Quantitative thresholds for acceptable miss rates in continuous pest-trapping are not yet standardized in the literature, and long-term field deployment will be needed to calibrate this number against agronomic outcomes. For multi-node coordination and remote deployment, LoRa and GPS modules were also evaluated: the LoRa link maintained a packet loss rate below 1.2% over 500 m line-of-sight, and GPS localization accuracy was within ±10 m, supporting regional coordination and tracking in potential field-scale applications.

Overall, the experimental results demonstrate that the proposed embedded pest detection and trapping prototype achieves high inference throughput, reliable real-time control, and stable laboratory operation. These results support the feasibility of the proposed system for subsequent outdoor and larger-scale validation in precision pest management applications.

From an agronomic management perspective, the system’s rapid response and high laboratory closed-loop success rate are engineering indicators of how quickly localized actuation can be triggered after detection. Whether this responsiveness translates into agronomic benefit, that is, measurable reductions in pest pressure, crop damage, or pesticide use, depends on field-scale factors including pest dynamics, trap selectivity, multiday operation, and seasonal variation, and would require dedicated field trials to assess. These observations therefore motivate but do not substitute for such field-scale validation.

In addition to the laboratory experiments, preliminary outdoor use was conducted for informal functional observation. Under natural field conditions, the prototype remained operational and could complete the detection-attraction-actuation loop, with observable attraction and trapping events during operation. However, no standardized field measurements, control-group comparisons, or quantitative efficacy analyses were performed at this stage. These preliminary observations should therefore be interpreted only as qualitative indications of outdoor operability and practical applicability, rather than as evidence of field-scale pest-control effectiveness.

### 3.4 Simulation of multi-node deployment

**Figure 11:**
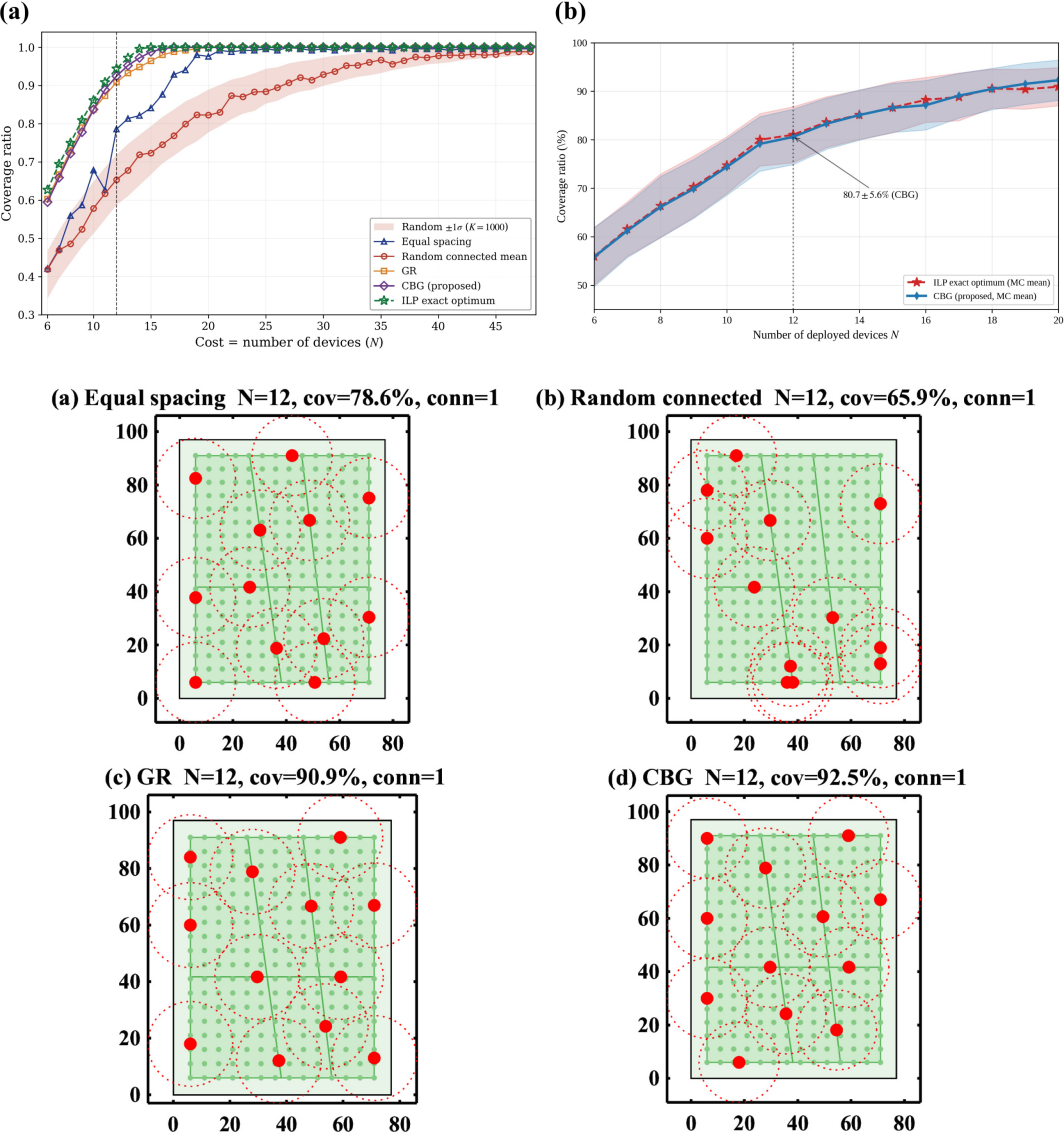
Multi-node deployment simulation results. **(a)** Noise-free cost–coverage curves for the placement strategies, with the ILP exact optimum overlaid as the proven upper bound; **(b)** Monte Carlo reevaluation of the same deployment family under the Section 2.3.3 field-realistic uncertainty model. **(c)** Spatial layouts at *N* = 12; red circles = devices and working radius, green points = evaluation locations; cov and conn are annotated per panel.

The effectiveness of deployment strategies was evaluated via simulation. **Figure 11a** plots the coverage ratio versus device budget (number of deployed units). We consider four strategies: equal spacing, random connected, GR, and CBG. Equal spacing places devices on a regular lattice over the target parcel (grid-based baseline), providing a simple, scalable reference for area coverage. Random connected samples locations uniformly at random and then enforces connectivity by adding relays or selecting only configurations that form a connected communication graph (random-placement baseline). GR connected follows the classical connected sensor-cover paradigm, iteratively adding the candidate that yields the largest marginal coverage gain while maintaining connectivity (Zangina et al., 2021). CBG is the proposed cover-bridge greedy variant introduced in the Methods section, which augments greedy selection with coverage balancing to reduce overlap and improve reach. The shaded band around the random-connected curve indicates the interquartile range (25%–75%) across repeated trials, reflecting variability due to stochastic placement. To benchmark against the proven optimum and assess robustness, **Figure 11b** reevaluates the same field under the field-realistic uncertainty model of Section 2.3.3 (*K* = 300 Monte Carlo realisations per budget). It compares CBG against the ILP exact optimum solved with HiGHS, which supplies the proven upper bound on coverage in the noise-free case.

A closer inspection of **Figure 11a** reveals clear differences among the deployment approaches. The equal spacing method shows moderate growth in coverage but suffers from oscillations due to geometric mismatch with irregular paddy boundaries. The random connected method performs worst overall, with limited and inconsistent coverage even when the number of devices increases, highlighting the inefficiency of uncontrolled placement. The GR connected approach achieves rapid coverage improvement and stabilizes near 90%, demonstrating its effectiveness in sequentially maximizing marginal gain while preserving network connectivity. The CBG consistently outperforms the others, achieving over 90% coverage with fewer devices.

While **Figures 11a, b** highlight the quantitative performance of the algorithms, **Figure 11c** provides representative layouts for equal spacing, random connected, greedy connected, and CBG when the number of devices is fixed at the threshold N = 12, determined by the minimum budget required to achieve the predefined coverage target (90%). Each subfigure displays the device positions as red markers, along with their effective working radii, enabling a direct comparison of how different algorithms allocate resources within the same field.

At the representative budget N = 12, the CBG layout attains a coverage of 92.5%, compared with 90.9% for GR, 78.6% for equal spacing, and 65.9% for random connected placement. The numerical margin over GR is modest. Under a Monte Carlo uncertainty analysis driven by the Section 3.3 hardware measurements, the mean CBG coverage at *N* = 12 decreases to about 80.7 ± 5.6%, while the connectivity probability remains 1.0 (**Figure 11b**). A perturbation-wise decomposition shows that this reduction is not a network-reliability failure: communication packet loss has no measurable effect on coverage, independent device failure reduces coverage by only about 4 percentage points, and all layouts remain fully connected, indicating that the mesh communication topology absorbs link and node faults. The dominant component is a geometric effect of GPS localization error relative to the 15 m attraction radius rather than a system failure; depending on how the measured ±10 m accuracy is mapped to the perturbation scale, the coverage lies in an approximately 81–85% range, and we report the conservative *σ* = 5 m case. This residual loss is recoverable through a modest deployment-density margin: increasing the budget from 12 to 18 devices restores coverage to ≥ 90% with full connectivity, which we therefore present as a deployment-planning guideline rather than a defect. A sensitivity sweep over working radius, communication radius, candidate spacing, and field geometry confirms that the strategy ordering and full connectivity are preserved, whereas the exact CBG–GR margin is small and discretization-dependent; the deployment study is therefore presented as a conceptual analysis rather than a definitive optimization claim. Since the field diagonal of about 124 m is well below the communication radius, the deployment results are insensitive to it and remain identical for communication radii of 200 m and 500 m. The ILP exact optimum for the coverage subproblem, solved with HiGHS (Huangfu and Hall, 2018), is shown in **Figure 11** as an upper-bound reference. Because this formulation maximizes coverage alone, without a connectivity constraint, the ILP optimum bounds what is achievable rather than providing a deployable layout, whereas CBG secures coverage and connectivity together. Under the field-realistic uncertainty model, however, CBG and the ILP layout become statistically indistinguishable in coverage (**Figure 11b**), because CBG spreads its devices more widely and is therefore less sensitive to GPS localization error than the tightly packed ILP layout. Equal spacing leaves systematic voids along edges and corners because a uniform lattice cannot adapt to irregular parcel boundaries. Random connected placement forms clusters with high overlap that wastes devices. GR alleviates these issues by selecting sites with maximum marginal gain under a connectivity constraint, yet its early seeds often lie mid-segment on ridges, which turns later selections into relays and leaves residual gaps near intersections and boundaries. In contrast, CBG starts with a dispersion-aware seeding phase to reduce overlap and expand coverage, followed by a bridging phase that selects nodes contacting multiple components to enforce connectivity with few extra devices. The result is a shorter network diameter and earlier reach of edge regions, so subsequent additions target true boundary gaps instead of redundant interior sites. Through this mechanism, CBG delivers the observed +1.6% improvement over GR at N = 12 and a stronger position on the cost–coverage curve.

Overall, the simulations confirm that deployment strategy strongly influences multi-node field coverage. Random or equal spacing strategies fail to provide reliable performance under limited budgets, whereas greedy optimization substantially improves both coverage and connectivity. The CBG method achieves the most favorable balance by combining dispersed high-gain seeding with component-aware bridging, which reduces redundancy while preserving robust communication. These results provide a practical basis for guiding deployment decisions in rice-field environments.

## 4 Discussion

This work presents a rice-field edge AI prototype for pest detection and trapping, evaluated through laboratory closed-loop testing and simulated multi-node deployment analysis. The discussion separates two distinct layers. The first is engineering feasibility, which the laboratory and simulation results address. The second is agronomic benefit, namely measurable reductions in pest pressure, crop damage, or pesticide use, which lies outside this study’s scope and would require dedicated field trials. The major engineering findings are summarized below:

1. A compact detection model named YOLOv13-M-G-P3 was developed by integrating the MAC2 module into the backbone and applying GhostConv at the shallow semantic level P3. The model achieved a mAP@0.5 of 96.1% in the PyTorch framework and outperformed baseline models such as YOLOv13 and YOLOv8 in both precision and recall. EigenCAM analysis further confirmed that the MAC2 module improved attention to insect contours and GhostConv enhanced spatial localization. These results demonstrate that the model provides both high accuracy and reliable interpretability while maintaining a compact size suitable for embedded deployment.

2. Robustness was systematically evaluated under synthetic fog, rain, and low-light degradations of varying severity. YOLOv13-M-G-P3 achieved the best performance in 6 out of 9 mAP cases, showing favorable comparative behavior under controlled perturbations. The model was, however, surpassed by YOLOv13-M-G-P34 and P345 under severe fog or rain, and by YOLOv13-M or the baseline under lightly degraded or dark conditions. These findings indicate that introducing GhostConv at P3 strengthens robustness in synthetic stress tests at the low semantic level, while excessive modification may compromise generalization ability.

3. A prototype system was implemented on Jetson Xavier NX with an ESP32-based peripheral platform and evaluated through a detection-to-control pipeline. On Tesla T4, the detector reached 59.4 FPS under FP16 TensorRT inference. On Jetson Xavier NX, the system sustained 44.5 FPS. The control subsystem delivered an average response time of 14.8 ms and achieved a laboratory closed-loop response success rate of 94.7%, confirming laboratory real-time feasibility and functional stability across all modules.

4. A simulation study was conducted to evaluate multi-node deployment in rice fields. Under fragmented 835 paddy conditions with connectivity constraints and a device budget of *N* = 12, the proposed CBG strategy reached 92.5% coverage. **Figure 11** shows that this deployment family stayed close to the ILP exact optimum on the tested field geometry in the nominal case and retained full connectivity under field-realistic uncertainty.

Despite the results obtained under laboratory and simulation settings, several limitations should be acknowledged. First, the study covers only a single rice-field scenario and a small, long-tailed dataset of 1,120 images, of which only 59 form the test subset and the dominant class accounts for 52.5% of instances. Because the overall mAP is shaped disproportionately by these dominant classes and each detector was trained once with a fixed seed, the reported values should be read as indicative rather than definitive. Narrow margins such as the +0.7% gain over YOLOv13 need confirmation by repeated runs, and wider applicability needs broader field conditions and more balanced data. Second, the degradation experiments in Section 3.2.3 were based on synthetically generated fog, rain, and low-light perturbations applied offline to clean images. These experiments provide controlled comparative stress-test evidence only and cannot substitute for independent field evaluation under real weather, illumination, blur, occlusion, and contamination conditions. Although the dataset contains naturally degraded scenes such as low-light and partial occlusion, we did not stratify detection performance on these subsets, because image-level condition metadata were not annotated at collection time. This stratification, together with on-device evaluation under live field conditions, is left to future work. Third, regarding field application, preliminary field use was conducted only for informal observation. While this suggested that the prototype could operate under outdoor conditions and that attraction and trapping events could occur during operation, no standardized field measurements, control-group comparisons, longitudinal pest-density monitoring, or quantitative efficacy analyses were performed. Therefore, these field observations should be interpreted only as qualitative evidence of operational feasibility and practical applicability, rather than as a rigorous assessment of field-scale pest-control effectiveness.

## 5 Conclusion

In conclusion, this work presents a rice-field edge AI prototype for pest detection and trapping, and evaluates its end-to-end feasibility through laboratory closed-loop testing and simulated multi-node deployment analysis. The laboratory results establish that the perception–actuation chain operates within real-time and connectivity constraints under controlled conditions; the simulation results establish the system-level multi-node deployment behaviour at this representative field scale. Outdoor robustness and agronomic efficacy, that is, measurable reductions in pest pressure, crop damage, or pesticide use, are not within the scope of this study and remain to be quantified through dedicated field trials and long-term agronomic experiments. Future work will pursue these field-scale evaluations together with on-device live-environment testing.

## Data Availability

The raw data supporting the conclusions of this article will be made available by the authors, without undue reservation.
